# Ferromagnetism on an atom-thick & extended 2D metal-organic coordination network

**DOI:** 10.1038/s41467-024-46115-z

**Published:** 2024-02-29

**Authors:** Jorge Lobo-Checa, Leyre Hernández-López, Mikhail M. Otrokov, Ignacio Piquero-Zulaica, Adriana E. Candia, Pierluigi Gargiani, David Serrate, Fernando Delgado, Manuel Valvidares, Jorge Cerdá, Andrés Arnau, Fernando Bartolomé

**Affiliations:** 1 Instituto de Nanociencia y Materiales de Aragón (INMA), CSIC-Universidad de Zaragoza, 50009 Zaragoza, Spain; 2 Departamento de Física de la Materia Condensada, Universidad de Zaragoza, E-50009 Zaragoza, Spain; 3 Centro de Física de Materiales CSIC/UPV-EHU-Materials Physics Center, Manuel Lardizabal 5, E-20018 San Sebastián, Spain; 4 Donostia International Physics Center, Paseo Manuel de Lardizabal 4, E-20018 San Sebastian, Spain; 5 IKERBASQUE, Basque Foundation for Science, E-48011 Bilbao, Spain; 6 Physics Department E20, Technical University of Munich, 85748 Garching, Germany; 7 Instituto de Desarrollo Tecnológico para la Industria Química (INTEC-UNL-CONICET), 3000 Santa Fe, Argentina; 8 Instituto de Física del Litoral, Universidad Nacional del Litoral (IFIS-UNL-CONICET), 3000 Santa Fe, Argentina; 9 ALBA Synchrotron Light Source, E-08290 Cerdanyola del Vallès, Spain; 10 Instituto de Estudios Avanzados IUDEA, Departamento de Física, Universidad de La Laguna, C/Astrofísico Francisco Sánchez, s/n, 38203 La Laguna, Spain; 11 Instituto de Ciencia de Materiales de Madrid, CSIC, Cantoblanco, 28049 Madrid Spain; 12 Departamento de Polímeros y Materiales Avanzados: Física, Química y Tecnología, Facultad de Química UPV/EHU, 20080 Donostia-San Sebastián, Spain; 13 Present Address: Instituto de Nanociencia y Materiales de Aragón (INMA), CSIC-Universidad de Zaragoza, Zaragoza, 50009 Spain

**Keywords:** Ferromagnetism, Surfaces, interfaces and thin films, Organic-inorganic nanostructures

## Abstract

Ferromagnetism is the collective alignment of atomic spins that retain a net magnetic moment below the Curie temperature, even in the absence of external magnetic fields. Reducing this fundamental property into strictly two-dimensions was proposed in metal-organic coordination networks, but thus far has eluded experimental realization. In this work, we demonstrate that extended, cooperative ferromagnetism is feasible in an atomically thin two-dimensional metal-organic coordination network, despite only ≈ 5% of the monolayer being composed of Fe atoms. The resulting ferromagnetic state exhibits an out-of-plane easy-axis square-like hysteresis loop with large coercive fields over 2 Tesla, significant magnetic anisotropy, and persists up to *T*_*C*_ ≈ 35 K. These properties are driven by exchange interactions mainly mediated by the molecular linkers. Our findings resolve a two decade search for ferromagnetism in two-dimensional metal-organic coordination networks.

## Abstract (web summary)

Despite having all the ingredients required for the formation of two-dimensional ferromagnetism, achieving such a magnetic state in atomically thin metal-organic coordination networks has proved to be a persistent challenge. Here, Lobo-Checa et al demonstrate 2Dferromagnetism in a self-assembled network, exhibiting coercive fields over 2 Tesla and a Curie temperature of 35K.

## Introduction

The formation of extended two-dimensional (2D) magnetic order has long been an active quest in condensed matter physics. In two dimensions, the Mermin-Wagner theorem precludes the formation of isotropic ferromagnetic order when mediated by short-range exchange interactions at finite temperatures^1^. This initially limited the investigation and exemplary cases to bulk materials featuring dominant in-plane interactions^2,3^ and ultrathin inorganic layers supported on metallic surfaces^4–7^. It is only very recently that the first examples of pure (i.e. substrate decoupled), extended 2D-ferromagnetism (FM) were obtained by exfoliation of van der Waals crystals^8,9^. In these cases the Mermin-Wagner limitations were surmounted by the presence of significant magnetic anisotropy. This development immediately sparked widespread attention due to the many envisioned fundamental implications and extensive practical applications^10–12^. However, integrating layered van der Waals materials into devices has turned out to be extremely challenging as the lateral size and exact thickness of these layers is difficult to control^12^.

Earlier candidates to exhibit 2D-FM at the single-layer limit were metal-organic coordination networks (MOCNs) grown on metallic supports^13,14^. 2D-MOCNs a-priori contain all the essential ingredients to display 2D-FM: Selectable metallic centers providing non-zero atomic spins and incomplete quenching of the magnetic orbital moment (given their reduced point symmetry and chemical coordination)^15,16^, periodic spacing of these magnetic moments over the (non-magnetic) surfaces^17^, tunable lateral separation between adatoms by the synthetically variable organic linkers^18^, reduced electronic overlap of these metal centers with the substrate after 2D-MOCN formation^19^, and technically simple fabrication as they follow self-assembly protocols close to room temperature^20,21^. Despite these features, 2D-MOCNs at the single-layer limit have historically failed to explicitly exhibit 2D ferromagnetic remanence^13,14,17,22–27^. Many previous studies of 2D-MOCNs exhibited noticeable magnetic anisotropies, but at the single-layer limit neither spontaneous magnetization nor remanence were ever observed^13,14,17,22–27^. Moreover, the coupling among these metal centers is generally interpreted in terms of superexchange mechanisms through the organic ligands and less so by surface electrons^28^.

In this work, we study the magnetism of a single, atomically thin 2D-MOCN consisting of Fe atom centers and 9,10-dicyanoanthracene (DCA) molecular linkers forming a mixed honeycomb kagome lattice on Au(111). We take advantage of the monodomain and extended character of this network in a multitechnique approach. Particularly, we combine scanning tunneling microscopy and spectroscopy (STM/STS), low energy electron diffraction (LEED), X-Ray absorption spectroscopy (XAS), X-Ray magnetic circular dichromism (XMCD), and X-ray photoemission spectroscopy (XPS) techniques. We present clear evidence of long-range ferromagnetic order in a 2D-MOCN with a Curie temperature (*T*_*C*_) of ≈ 35 K. The ferromagnetic state displays very strong out-of-plane (OOP) magnetic anisotropy and has a square hysteresis loop with a coercive field of ≈ 2.1 T. The XMCD orbital sum rule yields a maximal unquenched OOP orbital magnetic moment for Fe centers of $\langle {m}_{z}^{l}\rangle \approx 2\,{\mu }_{B}$. The magnetization as a function of temperature through the FM phase transition falls close to the honeycomb 2D Ising model involving strong uniaxial anisotropic magnetic centers. These results strongly differ from those obtained for the inorganic system formed in absence of DCA molecules, which results in an array of Fe clusters on the Au(111) surface. We make use of first-principles density functional theory calculations to explain the observation of FM at finite temperature in this 2D-MOCN system, which exhibits a large single ion anisotropy at the Fe atoms and a significant exchange interaction across the molecular linkers with a limited contribution through the underlying substrate.

## Results and discussion

### Spatial and electronic structure of the 2D-MOCN

**Fig. 1:**
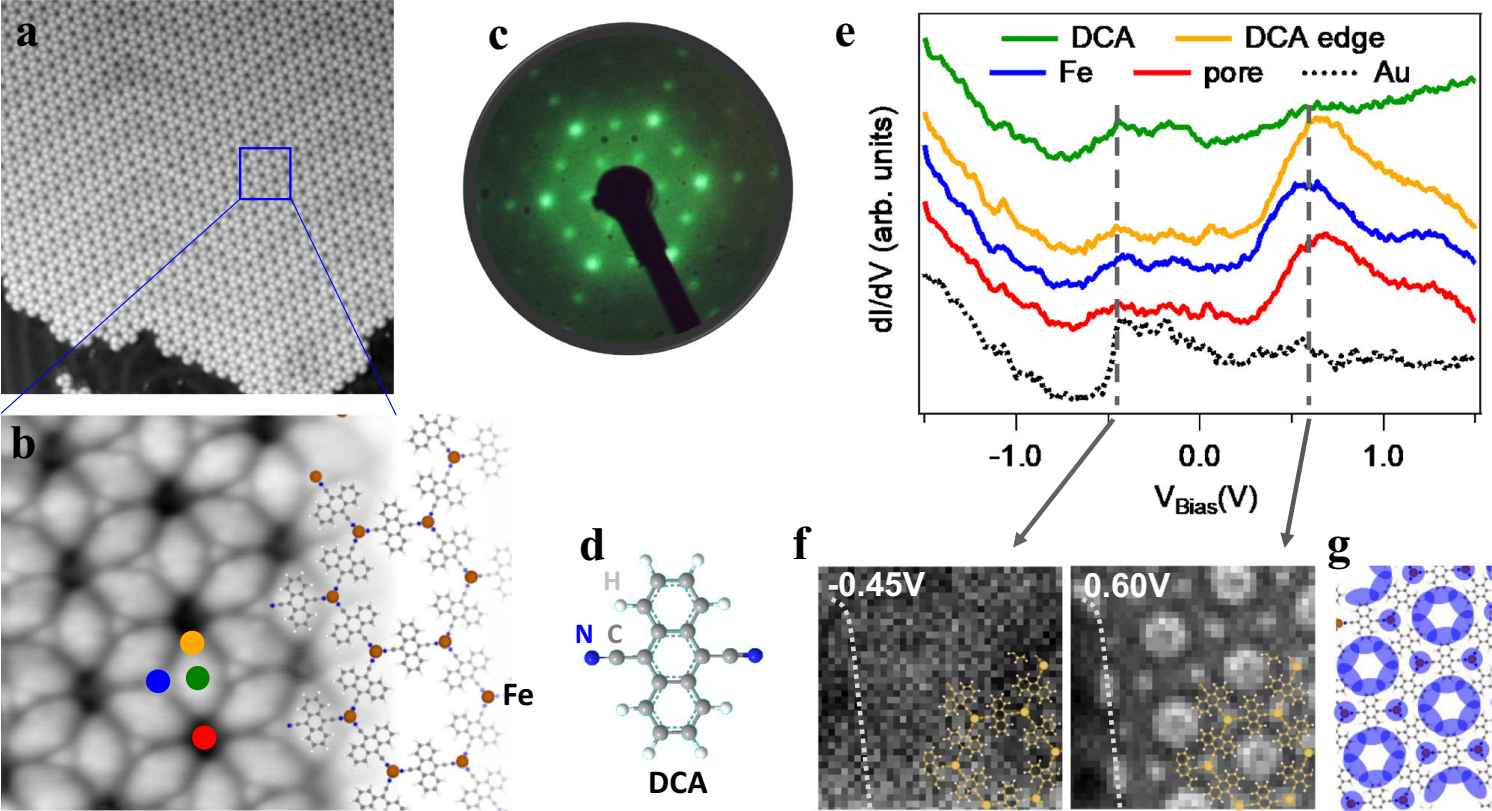
Atomic and electronic structure of the Fe-DCA/Au(111) network. Overview (**a**) and close-up (**b**) images of the 2D-metal-organic lattice. A model of the MOCN structure is overlaid in **b** with the molecules forming a kagome substructure and the Fe centers (orange-brown spheres) a honeycomb sublattice. **c** LEED pattern of this network (at 19 eV and room temperature) displaying a $(4\sqrt{3}\times 4\sqrt{3})$R30° structure with respect to the underlying substrate. **d** Stick-ball model of 9,10-dicyanoanthracene (DCA) molecule (C in gray, N in blue and H in white). **e** STS averaged spectra extracted from a dI/dV grid at the positions marked as circles of the same color in **b**. A network (collective) electronic state is located around 0.60 eV. **f** dI/dV isoenergetic maps at the energies indicated by the vertical discontinuous lines in **e**. The gray dotted line on the left of the maps marks the edge of an island. **g** Cartoon of the dominant spatial distribution (dark blue) of the network state identified at 0.60 eV. STM parameters: **a** 50 × 50 nm^2^, 100 pA; −100 mV; **b** 6 × 6 nm^2^, 20 pA; −1 V; **e**, **f** Setpoint 100 pA, −1V, *V*_*rms*_ = 15.8 mV, *f*_*osc*_ = 817 Hz).

The self-assembled Fe-DCA lattice is formed by sequential deposition of DCA molecules and Fe atoms on Au(111). Prior to the metal evaporation, the molecules form compact islands (see Supplementary Fig. S1) that evolve into the open network under a stoichiometric Fe:DCA relation of 2:3 (see Supplementary Fig. S2 for the effects of deviation from this proportion). The network is perfected after a mild annealing at 373 K for 10 min, generating large and monodomain network islands, similar to Cu-DCA/Cu(111)^29^. Figure 1a shows a typical overview of this 2D-MOCN, where the Fe centers form a honeycomb array and the DCA linkers a Kagome sublattice (cf. Fig. 1b). This network does not destroy the herringbone reconstruction of the underlying Au(111), suggesting a weak surface-MOCN interaction^17^. The analysis of the LEED patterns (see Fig. 1c and Supplementary Fig. S3e, f) show a hexagonal network with $(4\sqrt{3}\times 4\sqrt{3})$R30° structure with respect to the underlying substrate, resulting in unit vectors of 2 nm with first Fe neighbors distanced by 1.15 nm.

The electronic structure of this 2D-MOCN is obtained by means of dI/dV grids from which STS spectra at selected network locations (Fig. 1e) and dI/dV maps (Fig. 1f) are extracted. These STS spectra clearly differ from the uncoordinated DCA (cf. Supplementary Fig. S1). Two broad features call for our attention: The first, in the occupied region close to the Shockley state onset position (≈− 0.50 V), and the second at the unoccupied region centered at 0.60 V. In the dI/dV map at −0.45 V this first state is rather featureless both on the metal and throughout the network, revealing a substrate origin. This is supported by the fact that no distinct confined state could be detected at the MOCN nanocavity centers, which further supports the weak surface-network interaction. Contrarily, the dI/dV map at 0.60 V shows distinct features throughout the network, primarily at the DCA edges and at the metal centers, as sketched in Fig. 1g. This has been identified as the fingerprint of an extended network multi-band in the related Cu-DCA/Cu(111) system^29^.

### Magnetic characterization

**Fig. 2:**
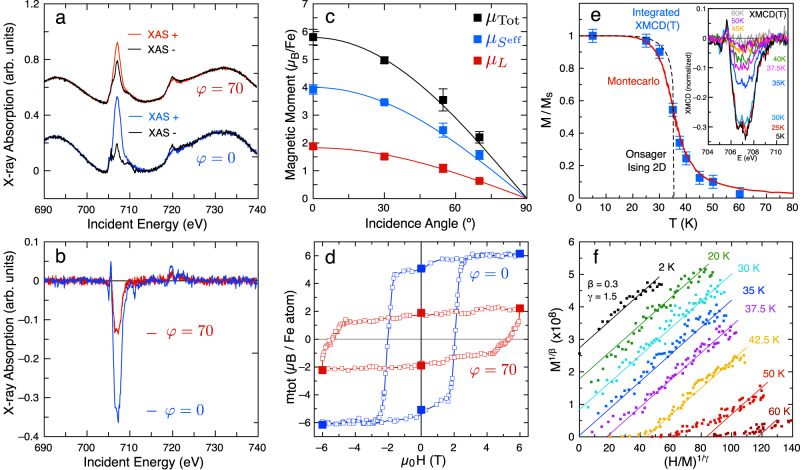
Experimental demonstration of 2D-Ferromagnetism of the Fe-DCA network on Au(111). **a** XAS (displaced vertically for clarity) and corresponding (**b**) XMCD spectra acquired with circularly right (*I*^+^) and left (*I*^−^) polarized X-ray light for normal (0°) and grazing (70°) incidence at the *L*_2,3_ edges of Fe. The Fe *L*_2,3_ XAS sits on top of the Au EXAFS background. A strong out-of-plane magnetic anisotropy is evidenced by inspection. **c** Angular dependence of the orbital (red), effective spin (blue) and total (black) magnetic moments obtained from the sum rules. The lines follow a cosine relation: ${\mu }_{\kappa }^{\varphi=0}\cdot\cos (\varphi )$ with *κ* = *L*, *S*_eff_, and Total. **d** Hysteresis loops (open symbols) obtained at the *L*_3_ edge of Fe at normal (*φ* = 0°, blue) and grazing (*φ* = 70°, red) incidence. The solid symbols are the result of applying the sum rules to the XAS and XMCD spectra obtained at remanence after conveniently cycling the field from ±6 T to zero for both incidence angles. **e** Integrated area (normalized to the saturation value) of the *L*_3_ XMCD main peak measured in normal incidence under low fields (*H* = 0.1 T) as a function of temperature from *T* = 5 K up to 60 K. The original XMCD data are shown in the inset. The Onsager 2D Ising analytical solution (dashed line) and a Monte Carlo simulation (red line), both for a honeycomb lattice are shown, satisfactorily describing the experimental data for *J* ≈ 2 meV. The experiment was performed crossing *T*_*C*_ heating up and then cooling down, exhibiting reversibility. Indeed, such *T*_*C*_ reversibility was reproduced on a second Au(111) substrate under *H* = 0.05 T (cf. Supplementary Fig. S6). **f** Modified Arrott-Noakes plot of isotherms with *β* = 0.30 and *γ* = 1.51 corresponding to a long-range 2D-Ising model^44^.

Our results evidence perfect crystalline quality and collective electronic states in this 2D-MOCN. Therefore, its magnetic properties can be unveiled using spatially averaging synchrotron-based techniques. A true magnetic signal probed by XAS and XMCD requires that we prevent Fe cluster formation (cf. Supplementary Figs. S2 and S3). Thus, we target untraceable Fe undercoverage samples exclusively leading to single metal centers surrounded by three N atoms (from different cyano groups) which results in a local three-fold symmetric arrangement (*C*_3*v*_). This is a single layer MOCN system, so interlayer coupling is discarded due to the non-magnetic character of the underlying Au substrate. Figure 2a and b show XAS and XMCD spectra respectively, acquired at the *L*_2,3_ edges of Fe at normal (*φ* = 0°) and grazing (*φ* = 70°) incidence (see Supplementary Information (S.I.) for experimental details). Both XAS and XMCD show narrow peak contributions, which are reminiscent of systems featuring monodispersed Fe atoms on surfaces^30,31^, or embedded in other 2D-MOCNs^17^, or forming part of molecules^15^. Indeed, the spectra clearly suggest a Fe(II) oxidation state (see for example ref. ^32^). To further confirm the absence of Fe cluster formation in the 2D-MOCN, we directly deposit the same amount of Fe on the clean Au(111) substrate (without DCA molecules) and measure Fe *L*_2,3_ XAS and XMCD under identical experimental conditions. The Fe/Au(111) results are shown in Supplementary Fig. S4. Distinctly different XAS and XMCD spectra are obtained compared to the ones of Fig. 2a which exhibit the typical smoother and broader metallic Fe *L*_2,3_ lineshapes^31,33^. These spectra evidence that Fe adatoms are assembled into small clusters nucleating at the herringbone elbows (see Supplementary Fig. S2a)^34,35^. A direct inspection of Fig. 2b reveals a strong OOP anisotropy for our 2D-MOCN. Identical results have been obtained in two different XAS/XMCD experimental runs, using different Au(111) crystals as substrate (cf. Supplementary Figs. S5 and S6). Despite a common OOP character, the Fe clusters in the Fe/Au(111) sample show a considerably smaller anisotropy than that found on the MOCN, and their *L*_3_ vs *L*_2_ branching ratio (relative peak intensities) is significantly lower than for the Fe-DCA network, evidencing a much smaller Fe orbital moment. Note that the Fe clusters are not fully comparable to the Fe centers in the 2D-MOCN because their geometry and chemical environments are different. Particularly, the coordination differs not only laterally, but also affects its vertical interaction with the substrate.

We can quantitatively determine the orbital (${\mu }_{L}^{z}$) and effective spin (${\mu }_{S}^{{{{{{{{{\rm{eff}}}}}}}}}_{z}}={\mu }_{S}-7{\mu }_{T}^{z}$) magnetic moments for the 2D-MOCN and the Fe clusters by using the X-ray magnetic dichroism sum rules^36,37^ (described in the Supplementary Section S3) with the XAS and XMCD spectra acquired at 4 different incidence angles (*φ* = 0°, 30°, 54. 7°, and 70°). The spectra were obtained in every case with the magnetic field parallel to the beam direction, such that XMCD yields the projection of the Fe magnetic moment along the direction of the applied field, *m*(*φ*) = **H** ⋅ **m**/*H*. We use the nominal number of holes in the 3*d* band for Fe(II) (*n*_*h*_ = 4) in these sum rules. As evidenced in Fig. 2c, ${\mu }_{L}^{z}$ and ${\mu }_{S}^{{{{{{{{{\rm{eff}}}}}}}}}_{z}}$ are strongly anisotropic, being much larger when the applied field is perpendicular to the 2D-MOCN plane. The obtained values in normal incidence are ${\mu }_{L}^{z}=1.88\pm 0.02\,{\mu }_{{{{{{{{\rm{B}}}}}}}}}$ and ${\mu }_{S}^{{{{{{{{{\rm{eff}}}}}}}}}_{z}}=4.02\pm 0.04\,{\mu }_{{{{{{{{\rm{B}}}}}}}}}$, consistent with a Fe(II) *d*^6^ high-spin configuration with L = 2 and S = 2, carrying a large orbital moment $\langle {m}_{z}^{l}\rangle \approx 2{\mu }_{{{{{{{{\rm{B}}}}}}}}}$, which is responsible for the large magnetic anisotropy. Such high spin configuration agrees with the 2^+^ state of the Fe 2*p* extracted from our XPS data (see Supplementary Section S4 and Supplementary Fig. S7). Similar cases of *d*^6^ high-spin strongly uniaxial angular momenta have been observed, such as single Fe(II) ions atop the nitrogen site of the Cu_2_N lattice^38^ and Co(III) in one-dimensional cobaltate Ca_3_Co_2_O_6_^16^.

The four incidence angles graphed in Fig. 2c were selected to allow direct separation of the isotropic spin moment, *μ*_*S*_, from the dipolar term $-7{\mu }_{T}^{z}$. In particular, if the magnetic moments rotate with the field as the angle-dependent experiment is performed, at the so-called magic angle *φ* = 54. 7°, the intra-atomic dipolar contribution ${\mu }_{T}^{z}$ cancels, allowing a direct measure of *m*_*S*_^15,39^ (see Supplementary Section S3). However, this is not the case in Fe-DCA/Au(111) since we find that both ${\mu }_{L}^{z}(\varphi )$ and ${\mu }_{S}^{{{{{{{{{\rm{eff}}}}}}}}}_{z}}(\varphi )$ vary as $\cos (\varphi )$. Such behavior can only be rationalized if the magnetic moments stay perpendicular to the 2D-MOCN surface even for the extreme case of *φ* = 70° and applied fields of 6T (see Supplementary Fig. S8), thereby evidencing a robust Ising character with OOP quantization axis.

Such marked OOP robustness should stand out when recording hysteresis loops. Therefore, we acquire the magnetization curves at *T* = 3 K by measuring the XMCD intensity at the fixed photon energy of the *L*_3_-edge as a function of the applied magnetic field for normal (*φ* = 0°) and grazing (*φ* = 70°) incidence (see Fig. 2d). Remarkably, the 2D-MOCN presents a square-like open hysteresis loop with a huge coercive field value ( ≈ 2.1 T out-of-plane and ≈ 5.4 T at *φ* = 70°) and a remanence slightly above 80% of the saturation value. Full zero field XMCD spectra at *φ* = 0°, and 70° were measured directly after saturation with *μ*_0_*H* = ± 6 T, with sum-rules yielding the values shown as solid symbols in Fig. 2d, consistently scaling with the hysteresis loop curve displayed. It is worth noting that the *φ* = 0° hysteresis loop can be calculated from the *φ* = 70° one (and viceversa), by simply scaling the applied field as ${H}_{{0}^{\circ }}={H}_{7{0}^{\circ }}\cos (7{0}^{\circ })$ and the magnetization as ${M}_{{0}^{\circ }}={M}_{7{0}^{\circ }}/\cos (7{0}^{\circ })$ (see Supplementary Fig. S8). In other words, the OOP component on the hysteresis loops are identical for all measured angles when projecting the applied magnetic field in that direction, evidencing once more the intense uniaxial character of the magnetic moments of Fe(II) in this 2D-MOCN.

To determine whether the open hysteresis loop reflects a slow relaxation single-atom process^40^ or a truly 2D ferromagnetic cooperative phase transition, we measure low field (*μ*_0_*H* = 0.1 T) XMCD curves as a function of temperature. The spectra at the *L*_3_ edge are shown in the inset of Fig. 2e, where we observe a sudden reduction of the XMCD signal occurring around *T*_*C*_ ≈ 35 K. This critical temperature is evident in Fig. 2e when plotting the absolute value of the integrated area of the XMCD main peak at the *L*_3_ edge (from 705 to 712.5 eV). The 2D Onsager’s analytical solution (at zero field)^41^ scaled for *T*_*C*_ = 35 K is shown for comparison, yielding an exchange interaction of $J=[{T}_{C}\cdot {k}_{B}\cdot \log (\sqrt{3}+2)]/2\approx+1.98$ meV. A Monte Carlo simulation of the spontaneous magnetization under this low field (*μ*_0_*H* = 0.1 T) with that particular exchange interaction constant on a simple honeycomb network of Ising spins with nearest neighbor interactions quite satisfactorily fits our experimental data. Note that this exchange constant is considerable when compared with those found in other single-layer 2D-MOCNs (*J* ≤ 0.27 meV)^17,22,23,42^.

To further demonstrate that a cooperative ferromagnetic phase transition takes place in our 2D-MOCN we used the so-called modified Arrott-Noakes plots^43^, which allow to obtain from the isothermal XMCD magnetization curves the magnetization *β* and susceptibility *γ* critical exponents, while determining the critical temperature. The modified Arrot-Noakes plot displayed in Fig. 2f shows the expected temperature-independent slope of the curves at higher fields, neatly showing the cooperative character of the FM below *T*_*C*_ ≈ 35 K (whose curve satisfactorily crosses the origin) for *β* = 0.30 and *γ* = 1.50. However, given the XMCD signal to noise ratio, we estimate the uncertainty in the determination of the critical exponents not better than ± 15%. The obtained exponents are not coincident with the canonical 2D Ising model (*β* = 1/8, *γ* = 7/4), but are compatible with other well-established cases of strongly uniaxial 2D systems with long-range interactions, such as URhAl (*β* = 0.287 and *γ* = 1.47)^44^. Considering the low amount of Fe centers present in the system (≈5% of a monolayer), the experimental determination of these critical values, even with this large uncertainty, is a remarkable achievement in itself^45^. In short, all our XMCD datasets present this 2D-MOCN as an archetype example of a two-dimensional ferromagnet at the single-layer limit.

It is worth noting that Fe magnetic moments are strongly dissimilar when comparing Fe-DCA/Au(111) and Fe/Au(111) samples (cf. Fig. 2b and Supplementary Fig. S4a and Supplementary Table S1). Remarkably, the hysteresis loops measured on the Fe clusters (see Supplementary Fig. S4d) are rather similar at *φ* = 0° and 70° and do not reach saturation at the highest accessible external field (6 T). Moreover, the butterfly-shape hysteresis found in grazing incidence (closure at 0 T) of Fe/Au(111) is characteristic of paramagnetic (or superparamagnetic) systems with long relaxation times^46,47^. Such differences in the Fe magnetic moments are related to a change of oxidation state of the Fe atoms associated with the 3*d* orbital occupation of the Fe atoms. In the case of the Fe-DCA/Au(111) network, the Fe atom is in a 2+ oxidation state, whereas in the case of Fe/Au(111) it is in a 0 oxidation state (metallic) (see Supplementary Section S4 and Supplementary Fig. S7). The 2+ state is mostly determined by the mechanism of charge transfer from Fe to DCA in Fe-DCA/Au(111), although there is also a sizable charge transfer from the Fe-DCA overlayer to the Au(111) surface (discussed below). Importantly, such a Fe2^+^ state is also supported by semi-empirical multiplet calculations based on the XAS/XMCD spectra (see Supplementary Fig. S9).

### Theory and discussion

**Fig. 3:**
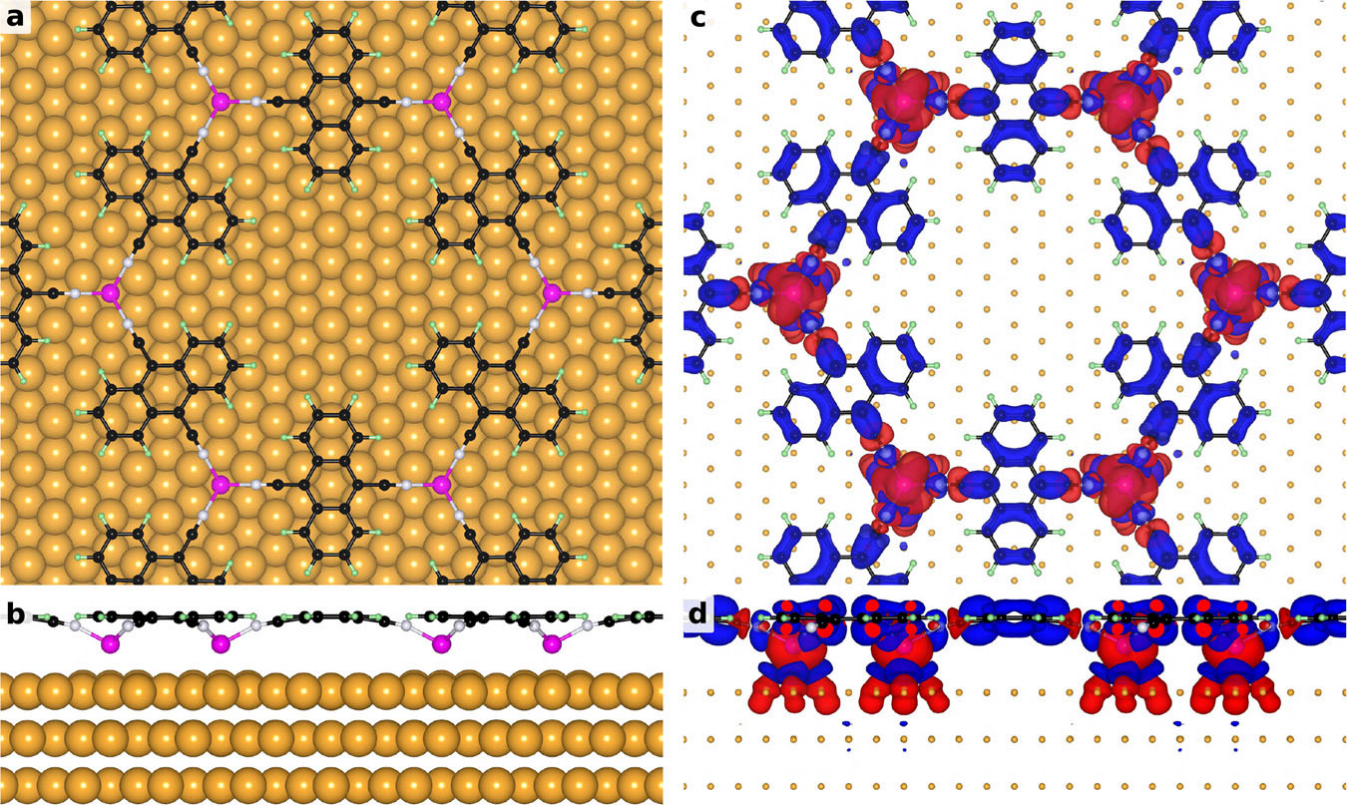
DFT calculations of the Fe-DCA/Au(111) system. Top (**a**) and side (**b**) views of the 2D-MOCN optimized structure with Au, Fe, C, N and H atoms in gold, purple, black, white, and green colors, respectively. The corresponding spin density isocontours [red (blue) denotes the majority (minority) spin component] are shown in panels **c** and **d**. This spin density reveals a predominant coupling between Fe magnetic moments through the DCA ligands and a sizable spin polarization of the Au atoms below the Fe atoms. Note that the absence of significant spin density connecting the Au atoms evidences a limited coupling mediated by the metal surface.

To shed light on these results we perform first-principles density functional theory (DFT) calculations (details in Supplementary Section S2). We start by structurally optimizing the Fe-DCA/Au(111) system using its experimentally determined $(4\sqrt{3}\times 4\sqrt{3})$R30° periodicity and obtain a non-planar MOCN geometry, shown in Fig. 3a, b (see Supplementary Section S2 for the crystal structure details). The vertical distortion affects the cyano groups that bend downwards to the Fe centers. Such buckled geometry has been observed in many other single-layer 2D-MOCN on noble-metal surfaces^13,29,48,49^. Independently of this buckled geometry, the MOCN retains its two-dimensional character with respect to the magnetic properties of interest since the Fe atoms are coplanar, very much like the transition metal atoms in other 2D-systems, e.g., CrI_3_. Importantly, the shortest Fe-Au distance is about 2.8 Å (see Supplementary Table S5), which is more than 10 % larger compared to the optimized Fe-honeycomb/Au(111) system (i.e. the one relaxed without DCA; see Supplementary Section S5). Thus, the Fe-Au hybridization is quite limited in Fe-DCA/Au(111), as anticipated experimentally. Such hybridization reduction related to the separation of the coordination atom from the substrate is common to other 2D-MOCNs^19,50^.

Total-energy calculations of this Fe-DCA/Au(111) structure result in a ferromagnetic isotropic exchange coupling constant of *J* ≃ 0.7–1.3 meV (Hubbard *U*_*eff*_ parameter dependent, see Supplementary Table S2). This exchange coupling strength appears to be an order of magnitude weaker when compared to the free-standing planar Fe-DCA system, as discussed in detail in Supplementary Section S5. Noteworthy in this context, is that the buckling of the MOCN induced by the Au(111) substrate makes the Fe-N bond length to be 6–8% longer than in the free-standing Fe-DCA case. This translates into a significantly smaller induced spin polarization in the DCA ligands with respect to that in the free-standing MOCN case, which is consistent with the weaker exchange coupling between the Fe centers. Incidentally, the spin moment of the DCA molecules is opposite in sign to that of Fe, revealing AFM coupling between the Fe centers and DCA ligands, clearly seen in the spin density isosurfaces (Fig. 3c, d). It should be said, however, that the isotropic exchange coupling *J* between Fe atoms in Fe-DCA/Au(111) is of the same order of magnitude as that between Cr atoms in CrI_3_^51^.

Furthermore, for Fe-DCA/Au(111), we find a positive magnetic anisotropy energy of *E*_*a*_ ≃ 0.6 meV per Fe atom (see Supplementary Section S2), indicating an OOP easy magnetization axis, contributions from the single ion anisotropy *D* and anisotropic exchange interaction *λ* being about 2*DS*^2^ ≃ 1.5 meV and 3*λS*^2^ ≃ − 0.25 meV (i.e., *E*_*a*_ ≃ *DS*^2^ + 3*λS*^2^/2^51^), respectively. Since DFT tends to underestimate the *E*_*a*_ value^52^, we have also performed multiplet calculations within a many-body approach based on a point-charge crystal field and find a much larger value of 8.5 meV (Supplementary Section S2 and Supplementary Fig. S10). Despite its quantitative limitations, DFT clearly confirms that the system is a ferromagnet with an OOP orientation of the local magnetic moments of 3.7*μ*_*B*_ (*S* = 2 state), which nicely matches our experimental results (compare Supplementary Tables S1 and S2 in Supplementary Section S6) and the values obtained from the multiplet calculations. Note that magnetic coupling has been reported in other single-layer 2D-MOCNs directly grown on metals^13,14,17,22–27^. However, in contrast to what is reported here, no open hysteresis loops were detected in any of the previously studied single-layer 2D-MOCNs at the atom thickness limit.

An intriguing question remaining is the definition of the exchange channels that drive the system into such collective magnetic state. From the spin density isosurfaces (Fig. 3c, d) it is seen that there is significant spin density delocalization in the DCA ligands connecting Fe metal centers. Not only the cyano groups, but also the anthracene backbones of DCA appear to be polarized, except for hydrogen atoms, which do not form part of the superexchange coupling path between Fe centers. On the contrary, the spin polarization is only induced on those Au atoms that are the Fe atom’s nearest neighbors, which is more clearly seen in the side view (Fig. 3d). Thus, the spin density in Au(111) is quite localized. The fact that no appreciable spin density connects two neighboring Fe centers of Fe-DCA through the Au(111) surface hints to a minor role of the substrate in mediating the exchange coupling between the Fe centers. Therefore, the superexchange via organic ligands dominates in Fe-DCA/Au(111), in agreement with previous works on 2D-MOCN^17,53,54^. However, we do expect some limited coupling mediated by the metal surface, *J*_*substr*_, caused by the weak (but still non-negligible) Fe-Au hybridization^55–58^. Regrettably, such coupling mediated by the substrate cannot be separated from that through the ligands in the full Fe-DCA/Au(111) calculation. Therefore, we perform first-principles calculations where we remove the DCA molecules from the optimized system, while keeping the honeycomb Fe array and the underlying Au(111) substrate fixed. Crucially, in this system we fix the Fe-3*d* manifold occupations to those of Fe in Fe-DCA/Au(111), which is done using the occupation matrix control method. As we discuss in detail in Supplementary Section S5, in spite of being in different chemical environments (Fe-honeycomb-on-Au(111) vs Fe-DCA/Au(111)), the Fe atoms have nearly the same magnetic state. Moreover, the fact that the lateral separation between the Fe atoms as well as their adsorption height above Au(111) turn out to be exactly the same, gives us grounds to use the Fe-honeycomb-on-Au(111) system to estimate the scale of the strength of *J*_*substr*_ in Fe-DCA/Au(111). In this way, we find that the substrate contribution to the isotropic exchange is limited to only *J*_*substr*_ ≃ 0.03 − 0.07 meV (ferromagnetic coupling). Note that this approximation can only provide an order of magnitude estimate because in our calculations the Fe atoms in Fe-honeycomb-on-Au(111) and Fe-DCA/Au(111) are not in an identical state (see Supplementary Section S5).

However, this does not mean that the Au(111) surface plays no role in the observed magnetism of the Fe-DCA MOCN grown on top. Indeed, after formation on the substrate, the network becomes buckled, thereby affecting the Fe-N bond length and hence the induced spin-polarization on the DCA, which in turn influences the main superexchange channel of the Fe-DCA/Au(111). Moreover, there is a charge transfer from the MOCN to the substrate (Supplementary Fig. S11) that affects the Fe-3*d* occupations (see Supplementary Section S5 and Supplementary Tables S4 and S5), defining the spin magnetic moment.

Summarizing the theoretical part, for this 2D-FM to occur in a MOCN, our first-principles calculations identify two necessary conditions: (i) the existence of hybrid bands (see Supplementary Fig. S11) with magnetic metal centers and organic linker orbital characters responsible for the ferromagnetic coupling between spin magnetic moments in the meV range, and (ii) a large magnetic anisotropy that translates into the opening of a gap in the spin wave excitation spectrum to overcome the Mermin-Wagner theorem^1^. These two key ingredients turn out to be truly remarkable in this Fe-DCA/Au(111) system, causing such finite temperature FM to emerge. Indeed, we find the same order of magnitude in *J* and roughly equal magnetic anisotropy as in the CrI_3_ monolayer (*J* = 2.2 meV and *E*_*a*_ = 0.65 meV^51^). Thus, it is rather unsurprising that our 2D-MOCN also presents a similar *T*_*C*_ as the one experimentally determined for this 2D-van der Waals ferromagnet (*T*_*C*_ = 45 K^9^). At this point, we should mention that a great effort is being done to progress and overcome the challenges involved in tuning the magnetic interactions in metal-organic solids^59,60^. Improving the design of magnetic metal-organic compounds is key to obtain fully functional organic ferromagnets^61,62^.

In conclusion, we have studied the structural, electronic and magnetic properties of the Fe-DCA/Au(111) system at its single-layer thickness. This 2D-MOCN exhibits delocalized electronic states with bucked geometry, but limited interaction with the substrate based on the prevalence of the herringbone reconstruction that sets the stage for prevailing electronic overlap between molecules and metal centers and a robust ferromagnetic ground state. Remarkably, we find open hysteresis cycles, an extraordinarily large uniaxial anisotropy, and phase transition behavior which translates into indisputable experimental evidence of a metal-organic 2D ferromagnet with a Curie temperature of T_*C*_ ≈ 35 K. The observed ${\mu }_{L}^{z}/{\mu }_{S}^{ef{f}_{z}} \sim 1/2$ value, consistent with Fe(II) high-spin *d*^6^ configuration (L = S = 2) is large enough to lead to a high uniaxial magnetic anisotropy (easy OOP magnetization direction). Our first-principles calculations confirm both the order of magnitude of the FM exchange coupling and the sign of the magnetic anisotropy that are necessary to explain the observed magnetic order at a rather high temperature. Importantly, the magnetic exchange constant found for this system is comparable to the highest reported for the ultrathin 2D-van der Waals ferromagnets^10–12^ and is certainly much higher than all other previous 2D-MOCNs studied at the single-layer limit^13,14,17,22–27,42^.

Our findings settle over two-decades of search for atom-thick 2D-FM in MOCNs, thereby representing a clear advance in the transversal fields of magnetism and surface science. Similarly to the seminal work of isolating a single layer CrI_3_^9^, we expect to boost the community’s interest in magnetic 2D-MOCNs and trigger follow-up theoretical and experimental work capable of leading to new ferromagnetic systems that exhibit even higher ordering temperatures with such ultra-low magnetic atom densities.

## Methods

The Methods section, containing experimental and theoretical details, is included in the Supplementary Information file.

### Supplementary information

Supplementary Information

Peer Review File

## Data Availability

The experimental data that support these findings are available from the corresponding authors upon reasonable request.

## Code availability

The theoretical codes that support these findings are available from the corresponding authors upon reasonable request.

## References

[1] Mermin ND, Wagner H (1966). Absence of Ferromagnetism or Antiferromagnetism in One- or Two-Dimensional Isotropic Heisenberg Models. Phys. Rev. Lett..

[2] Yamada I (1972). Magnetic properties of K_2_CuF_4_–a transparent two-dimensional ferromagnet–. J. Phys. Soc. Jpn..

[3] Miedema AR (1974). Heisenberg ferromagnetism in two dimensions: An experimental study. AIP Conf. Proc..

[4] Pietzsch O, Kubetzka A, Bode M, Wiesendanger R (2001). Observation of Magnetic Hysteresis at the Nanometer Scale by Spin-Polarized Scanning Tunneling Spectroscopy. Science.

[5] Pietzsch O, Kubetzka A, Bode M, Wiesendanger R (2004). Spin-Polarized Scanning Tunneling Spectroscopy of Nanoscale Cobalt Islands on Cu(111). Phys. Rev. Lett..

[6] Krause S, Berbil-Bautista L, Herzog G, Bode M, Wiesendanger R (2007). Current-Induced Magnetization Switching with a Spin-Polarized Scanning Tunneling Microscope. Science.

[7] Bickel JE (2011). Magnetic properties of monolayer Co islands on Ir(111) probed by spin-resolved scanning tunneling microscopy. Phys. Rev. B.

[8] Gong C (2017). Discovery of intrinsic ferromagnetism in two-dimensional van der Waals crystals. Nature.

[9] Huang B (2017). Layer-dependent ferromagnetism in a van der Waals crystal down to the monolayer limit. Nature.

[10] Gong C, Zhang X (2019). Two-dimensional magnetic crystals and emergent heterostructure devices. Science.

[11] Soriano D, Katsnelson MI, Fernández-Rossier J (2020). Magnetic Two-Dimensional Chromium Trihalides: A Theoretical Perspective. Nano Lett..

[12] Wang QH (2022). The Magnetic Genome of Two-Dimensional van der Waals Materials. ACS Nano.

[13] Gambardella P (2009). Supramolecular control of the magnetic anisotropy in two-dimensional high-spin Fe arrays at a metal interface. Nat. Mater..

[14] Carbone C (2011). Self-Assembled Nanometer-Scale Magnetic Networks on Surfaces: Fundamental Interactions and Functional Properties. Adv. Funct. Mater..

[15] Bartolomé J (2010). Highly unquenched orbital moment in textured Fe-phthalocyanine thin films. Phys. Rev. B.

[16] Leedahl B (2019). Origin of ising magnetism in Ca_3_Co_2_O_6_ unveiled by orbital imaging. Nat. Commun..

[17] Umbach TR (2012). Ferromagnetic coupling of mononuclear Fe centers in a self-assembled metal-organic network on Au(111). Phys. Rev. Lett..

[18] Schlickum U (2007). Metal-Organic Honeycomb Nanomeshes with Tunable Cavity Size. Nano Lett..

[19] Piquero-Zulaica I (2019). Surface state tunable energy and mass renormalization from homothetic quantum dot arrays. Nanoscale.

[20] Bartels L (2010). Tailoring molecular layers at metal surfaces. Nat. Chem..

[21] Dong L, Gao Z, Lin N (2016). Self-assembly of metal-organic coordination structures on surfaces. Prog. Surf. Sci..

[22] Abdurakhmanova N (2013). Superexchange-mediated ferromagnetic coupling in two-dimensional Ni-TCNQ networks on metal surfaces. Phys. Rev. Lett..

[23] Giovanelli L (2014). Magnetic coupling and single-ion anisotropy in surface-supported Mn-based metal-organic networks. J. Phys. Chem. C..

[24] Arruda LM (2020). Surface-orientation- and ligand-dependent quenching of the spin magnetic moment of Co porphyrins adsorbed on Cu substrates. Phys. Chem. Chem. Phys..

[25] Moreno D (2022). Engineering Periodic Dinuclear Lanthanide-Directed Networks Featuring Tunable Energy Level Alignment and Magnetic Anisotropy by Metal Exchange. Small.

[26] Umbach TR (2014). Site-specific bonding of copper adatoms to pyridine end groups mediating the formation of two-dimensional coordination networks on metal surfaces. Phys. Rev. B - Condens. Matter Mater. Phys..

[27] Faraggi MN (2015). Modeling ferro- and antiferromagnetic interactions in metal-organic coordination networks. J. Phys. Chem. C..

[28] Yosida, K. *Theory of Magnetism* (Springer-Verlag, 1996).

[29] Hernández-López L (2021). Searching for kagome multi-bands and edge states in a predicted organic topological insulator. Nanoscale.

[30] Gambardella P (2002). Localized Magnetic States of Fe, Co, and Ni Impurities on Alkali Metal Films. Phys. Rev. Lett..

[31] Pacchioni GE (2015). Multiplet features and magnetic properties of Fe on Cu(111): From single atoms to small clusters. Phys. Rev. B Condens. Matter Mater. Phys..

[32] Kowalska JK (2017). Iron *L*_2,3_ -edge X-ray absorption and X-ray magnetic circular dichroism studies of molecular iron complexes with relevance to the FeMoCo and FeVCo active sites of nitrogenase. Inorg. Chem..

[33] Boeglin C, Stanescu S, Deville JP, Ohresser P, Brookes NB (2002). In-plane magnetocrystalline anisotropy observed on Fe/Cu(111) nanostructures grown on stepped surfaces. Phys. Rev. B Condens. Matter Mater. Phys..

[34] Delga A (2011). Electronic properties of Fe clusters on a Au(111) surface. Phys. Rev. B.

[35] Ohresser P, Brookes N, Padovani S, Scheurer F, Bulou H (2001). Magnetism of small Fe clusters on Au(111) studied by X-ray magnetic circular dichroism. Phys. Rev. B.

[36] Thole BT, Carra P, Sette F, van der Laan G (1992). X-ray circular dichroism as a probe of orbital magnetization. Phys. Rev. Lett..

[37] Carra P, Thole BT, Altarelli M, Wang X (1993). X-ray circular dichroism and local magnetic fields. Phys. Rev. Lett..

[38] Rejali R (2020). Complete reversal of the atomic unquenched orbital moment by a single electron. npj Quantum Mater..

[39] Stöhr J, König H (1995). Determination of spin-and orbital-moment anisotropies in transition metals by angle-dependent X-ray magnetic circular dichroism. Phys. Rev. Lett..

[40] Donati F (2016). Magnetic remanence in single atoms. Science.

[41] Onsager L (1944). Crystal statistics. I. A two-dimensional model with an order-disorder transition. Phys. Rev..

[42] Blanco-Rey M (2018). Magnetic properties of metal-organic coordination networks based on 3*d* transition metal atoms. Molecules.

[43] Arrott A, Noakes JE (1967). Approximate equation of state for nickel near its critical temperature. Phys. Rev. Lett..

[44] Tateiwa N, Pospíšil JCV, Haga Y, Yamamoto E (2018). Critical behavior of magnetization in urhal: Quasi-two-dimensional ising system with long-range interactions. Phys. Rev. B.

[45] Barla A (2016). Design and performance of BOREAS, the beamline for resonant X-ray absorption and scattering experiments at the ALBA synchrotron light source. J. Synchrotron Radiat..

[46] Jiang S-D, Wang B-W, Sun H-L, Wang Z-M, Gao S (2011). An organometallic single-ion magnet. J. Am. Chem. Soc..

[47] Zhu Y-Y (2011). An enantiopure Fe^III^_4_ single-molecule magnet. Chem. Commun..

[48] Gao Z (2020). Design and Synthesis of a Single-Layer Ferromagnetic Metal-Organic Framework with Topological Nontrivial Gaps. J. Phys. Chem. C..

[49] Wang W (2013). Cooperative modulation of electronic structures of aromatic molecules coupled to multiple metal contacts. Phys. Rev. Lett..

[50] Piquero-Zulaica I (2022). Engineering quantum states and electronic landscapes through surface molecular nanoarchitectures. Rev. Mod. Phys..

[51] Lado JL, Fernández-Rossier J (2017). On the origin of magnetic anisotropy in two dimensional CrI_3_. 2D Mater..

[52] Baumann S (2015). Origin of Perpendicular Magnetic Anisotropy and Large Orbital Moment in Fe Atoms on MgO. Phys. Rev. Lett..

[53] Bellini V (2011). Propagation of spin information at the supramolecular scale through heteroaromatic linkers. Phys. Rev. Lett..

[54] Wegner D (2009). Tuning molecule-mediated spin coupling in bottom-up-fabricated vanadium-tetracyanoethylene nanostructures. Phys. Rev. Lett..

[55] Wahl P (2007). Exchange interaction between single magnetic adatoms. Phys. Rev. Lett..

[56] Meier F, Zhou L, Wiebe J, Wiesendanger R (2008). Revealing magnetic interactions from single-atom magnetization curves. Science.

[57] Zhou L (2010). Strength and directionality of surface ruderman–kittel–kasuya–yosida interaction mapped on the atomic scale. Nat. Phys..

[58] Girovsky J (2017). Long-range ferrimagnetic order in a two-dimensional supramolecular Kondo lattice. Nat. Commun..

[59] Park JG (2021). Magnetic ordering through itinerant ferromagnetism in a metal-organic framework. Nat. Chem..

[60] Park JG (2023). Permanent porosity in the room-temperature magnet and magnonic material V(TCNE)_2_. ACS Cent. Sci..

[61] Thorarinsdottir AE, Harris TD (2020). Metal-organic framework magnets. Chem. Rev..

[62] Perlepe P (2020). Metal-organic magnets with large coercivity and ordering temperatures up to 242°C. Science.

