# Supplementary material for: Ferromagnetism on an atom-thick & extended 2D metal-organic coordination network
Source: Nat Commun. 2024 Feb 29;15:1858. doi: 10.1038/s41467-024-46115-z (PMC10904770; doi:10.1038/s41467-024-46115-z)
Supplement: Supplementary file 1 — Supplementary Information [file 41467_2024_46115_MOESM1_ESM.pdf]

Supplementary Information:
Ferromagnetism on an atom-thick & extended 2D-metal-organic coordination network

Jorge Lobo-Checa, Leyre Hernández-López, Mikhail M. Otrokov, Ignacio Piquero-Zulaica, Adriana E. Candia, Pierluigi Gargiani, David Serrate, Fernando Delgado, Manuel Valvidares, Jorge Cerdá, Andrés Arnau, and Fernando Bartolomé

This document contains the following sections:

- S1. Experimental Methods.
- S2. Theoretical Methods.
- S3. XMCD sum rules.
- S4. Redox states determined by XPS.
- S5. Role of the Au(111) substrate in the magnetic properties of the Fe-DCA MOCN.
- S6. Supplementary tables
- S7. Supplementary figures.
- Supplementary references.

S1. EXPERIMENTAL METHODS

- Sample preparation.

All the experiments were performed under ultra-high vacuum conditions. The base pressure in the preparation chamber was below $5 \cdot 10^{-10}$ mbar. Au(111) single crystals were prepared by repeated cycles of Ar^+ sputtering at 1.5 kV and annealing at 750 K. DCA molecules were evaporated from a Knudsen cell by thermal evaporation at a rate of ≈ 0.03 monolayer per minute (ML/min) at room temperature (RT). Fe atoms were subsequently evaporated from an e-beam evaporator at a rate of ≈ 0.02 ML/min at RT. Finally an annealing up to 370 K was performed in all samples to improve the MOCN long-range order.

The full network ML coverage of DCA was calibrated by directly depositing the molecules on a Cu(111) sample. On this surface the Cu-DCA MOCN spontaneously forms at RT and saturates the surface with the 2D network [1]. Once the DCA evaporator was calibrated, the same deposition was done on Au(111) and the LEED was checked revealing the pattern shown in Fig. S1. To generate the Fe-DCA network we deposited minute amounts of Fe atoms, annealed at 370 K and checked the LEED at each evaporation step. We continued adding Fe until the LEED pattern eventually transformed into the pattern shown in Fig. S3.

Once the Fe and DCA evaporators were calibrated, we formed the Fe-DCA/Au(111) network on a single step. In the case of XAS and XMCD measurements, we took the precaution of reducing the Fe amount by 5% to avoid Fe clusters forming on the surface (compare Figs. S2 and S3).

- STM equipments.

The initial topography and the determination of the electronic properties of the 2D-MOCN were carried out at the Laboratorio de Microscopías Avanzadas (LMA) of the Universidad de Zaragoza using a low-temperature STM (LT-STM). This chamber has a base pressure better than $1 \cdot 10^{-10}$ mbar and operates at ~ 4 K. A W tip was used in all cases. All the voltages are referred to the sample. All presented images were acquired with constant current mode.

A second setup equipped with a RHK STM head was used at the BOREAS beamline in ALBA to judge the sample quality measured by XAS and XMCD. This equipment was operated at RT at a base pressure of $3 \cdot 10^{-10}$ mbar. We acquired topographic images in constant current mode, as shown in Fig. S3.

- XAS and XMCD setup.

X-ray absorption and dichroism experiments were performed at the Boreas beamline at ALBA synchrotron [2]. We did so in two different synchrotron runs with one year of time separation. The field and temperature used for XAS and XMCD spectra collection were $H = 6$ T and $T \sim 3$ K, unless otherwise stated. We measured at the Fe $L_{2,3}$ edge fixing the magnetic field with the direction of the incident light. The detection mode was total electron yield. The angle-dependent measurements were performed by rotating the sample about a vertical axis perpendicular to the synchrotron orbital plane, thereby varying the incidence angle φ between the X-ray beam (and therefore the mangetic field) and the substrate normal. To minimize experimental artifacts and reduce drift phenomena on the XMCD, we changed either the light helicity or the field direction during measurements. We increased the statistics by alternatively acquiring spectra for different helicities.

It is important to indicate that beam damage existed. Indeed, we destroyed this network within seconds when we illuminated the 2D-MOCN with the full collimated beam. When that happened the XAS and XMCD spectra showed different lineshapes to Fig. 2a,b and, remarkably, the hysteresis loops closed. We learned to avoid this beam damage by reducing the full intensity by a factor of roughly 100 (detuning both the beam focus and also the undulators). Although the beam damage was avoided (several hours timescale), we further prevented adquisition errors by continuously moving the spot on the sample surface (disc of 6.5 mm diameter) to continuously access non-illuminated fresh sample spots.

- XPS equipment.

X-ray Photoemission Spectroscopy was performed at the NanoPhysics Lab of the Centro de Física de Materiales in San Sebastian (Spain), with the help of Drs. Celia Rogero and Maxim Ilyn. A Phoibos photoelectron spectrometer combined with a non-monochromatized Al K_α X-ray source (16 mA, 12.5 kV) was used under a base pressure of $3 \cdot 10^{-9}$ mbar. The overall resolution of the instrument is ~0.9 eV. Importantly, the minute amounts of Fe (less than 5% of a ML) and DCA turned out to be within the detection limit of the instrument, resulting in very noisy signals (see Fig. S7). The individual spectra were consecutively acquired at RT and the acquisition times were above 1 hour per spectrum (roughly 8 hours per sample), which was restricted to avoid the

emergence of contamination traces in the carbon and oxygen CL lines.

S2. THEORETICAL METHODS

- Density Functional Theory (DFT) calculations.

Electronic structure calculations were carried out within density functional theory using the projector augmented-wave (PAW) method [3] as implemented in the VASP code [4, 5]. The exchange-correlation energy was treated using the generalized gradient approximation [6]. The Hamiltonian contained scalar relativistic corrections and the spin-orbit coupling was taken into account by the second variation method [7]. The energy cutoff for the plane-wave expansion was set to 400 eV. The Fe $3d$-states were treated employing the GGA+U approach [8] within the Dudarev scheme [9]. The $U_{\text{eff}} = 4, 5,$ and 6 eV values were used for our first-principles calculations. The atomic positions were optimized for each U_{eff} and then the magnetic ordering, magnetic anisotropy, and electronic structure were studied (see Table S2). It was found that neither the magnetic ground state nor the electronic structure change qualitatively upon such a variation of U_{eff}. In the cases where imposing particular occupation numbers of the Fe-$3d$ manifold was required (see Section S5 below), we used the occupation matrix control (OMC) method developed by Allen and Watson [10]. This was done in a two-step process, where the occupation constraint is first used to pre-converge the charge density and then a standard unconstrained DFT calculation for the desired Fe-$3d$ configuration is performed.

Fe-DCA/Au(111) was simulated within the supercell approach using the experimentally found $(4\sqrt{3} \times 4\sqrt{3})$R30° periodicity, where the Fe atoms of the MOCN were residing in the substrate's fcc hollow sites. The cells contained a vacuum layer of a minimum of 10 Å.

The free-standing Fe-DCA was fully optimized, i.e., lattice parameter and atomic coordinates corresponding to the minimal energy were found. For all U_{eff}, the lattice parameter of Fe-DCA is roughly $a = 20.5$ Å, which corresponds to Fe-Fe distance of 11.84 Å. A compressive strain of about 2.55 % is needed for its cell to match the experimentally found $(4\sqrt{3} \times 4\sqrt{3})$R30° Fe-DCA/Au(111) periodicity, as the optimized bulk lattice parameter of Au is 2.9153 Å. This strain is accomodated via loss of the Fe-DCA planarity. Three Au layers were used to simulate the Au(111) substrate, so that the Fe-DCA/Au(111) cell contained 224 atoms. The atoms of the lowermost Au layer were fixed during the structural relaxations, while the other two as well as

the MOCN were allowed to relax. The atomic coordinates were relaxed using a force tolerance criterion for convergence of 0.01 eV/Å. In all relaxations the 2D Brillouin zone was sampled with a $2\times2\times1$ $\overline{\Gamma}$-centered k-mesh. Using denser mesh eventually results into only ~ 7 % change of the exchange coupling constant, while significantly slows down the calculation. All of the total-energy (static) calculations where performed using the $7\times7\times1$ $\overline{\Gamma}$-centered k-mesh.

We consider the 2D Heisenberg model Hamiltonian with the out-of-plane easy-axis anisotropy:

$$H = -\frac{J}{2}\sum_{i,j}\mathbf{S}_i\mathbf{S}_j - \frac{\lambda}{2}\sum_{i,j}S_i^zS_j^z - D\sum_i(S_i^z)^2, \tag{S1}$$

where J is isotropic exchange coupling constant, λ, D are the parameters of the exchange anisotropy and single-site anisotropy, respectively, while i and j denote nearest neighbors within the layer. While J is obtained via a scalar relativistic DFT calculation of ferromagnetic and antiferromagnetic configurations, determining λ and D requires a relativistic calculation of the latter configurations for both in-plane and out-of-plane moment directions. Structure-wise, we have used a periodic supercell with two Fe atoms, i.e., one pair of interacting spins, each Fe atom with its three nearest neighbours, two of them in neighbour unit cells. This choice of supercell translates into an exchange coupling energy JS^2 per spin pair so we use the expression $J = \Delta_{A/F}/6S^2$ ($\Delta_{A/F} = E_{AFM} - E_{FM}$) to estimate the value of J.

The J values are presented in Table S2 for three different Hubbard U_{eff} parameters. The coupling is ferromagnetic in all cases, its strength being sensitive to the U_{eff} parameter. It is interesting to note that the isotropic exchange coupling between Fe atoms in the Fe-DCA/Au(111) is comparable to that between Cr atoms in CrI_3 [11, 12], being the distance between Fe atoms roughly two times larger than between Cr atoms. The explanation is that the strength of the superexchange coupling, mediated by the I orbitals in CrI_3 or by the DCA molecular orbitals in Fe-DCA/Au(111), does not depend so strongly on the size of the orbital, but rather on the overlap between the DCA and Fe orbitals. Indeed, a similar behavior has been found in one dimensional metal-organic polymeric chains of Co and Cr atoms with organic QDI ligands, with exchange coupling constants of the order of meV [13].

The magnetic anisotropy energy, $E_a = E_{diff} + E_d$, was calculated taking into account the total energy differences, $E_{diff} = E_{in-plane} - E_{out-of-plane}$, and the energy of the classical dipole-dipole interaction, E_d. For the E_{diff} calculation, a k mesh of $7\times7\times1$ points was chosen. To calculate

E_{diff}, the energies for three inequivalent magnetization directions [Cartesian x, y (in-plane) and z (out-of-plane)] were calculated and E_{diff} was determined as the difference $E_{in-plane}$ – E_z, where the $E_{in-plane}$ is the energy of the most energetically favorable in-plane direction of magnetization. The energy convergence criterion was set to 10^{-7} eV providing a well-converged E_{diff} (up to a few tenth of meV) while excluding "accidental" convergence. A cutoff radius of at least 20 microns was used to calculate dipole-dipole contribution E_d to the magnetic anisotropy energy. The obtained E_a values are included in Table S2. For all cases, the out-of-plane magnetization direction is favorable. Using the described 2D Heisenberg model [12], we find the single ion anisotropy $D = (\Delta E_{FM} + \Delta E_{AFM})/4S^2$ and the anisotropic exchange $\lambda = (\Delta E_{FM} - \Delta E_{AFM})/6S^2$. Here, $\Delta E_{FM} = E^x_{FM} - E^z_{FM}$ and $\Delta E_{AFM} = E^x_{AFM} - E^z_{AFM}$. For Fe-DCA/Au(111), $D \approx 0.19$ meV and $\lambda \approx -0.02$ meV, being both significantly smaller than the isotropic exchange with a higher contribution from single ion anisotropy.

To check the influence of the van der Waals (vdW) forces on the Fe-DCA/Au(111) structure and magnetism, we have performed the geometry optimization taking vdW corrections into account using the DFT-D3 scheme proposed by Grimme [14, 15]. Because of the additional attraction due to the vdW forces, the Fe-DCA MOCN gets closer to the surface as compared to the calculation without vdW corrections. As a result, the Fe atom and DCA backbone planes above the Au surface can now be found at ≈ 2.23 Å and ≈ 3.27 Å (compared to 2.5 Å and 3.9 Å without vdW corrections), respectively. At that distance, the Fe-Au and Fe-N bond lengths become 1.90 % and 2.44 % shorter and are equal to 2.06 Å and 2.79 Å, respectively (compared to 2.10 Å and 2.86 Å without vdW corrections), see Table S5. Notably, with these geometrical modifications we find that the Fe local magnetic moment undergoes practically no changes upon inclusion of the vdW forces during optimization, as this value is found to be 3.65 μ_B (without vdW corrections it is 3.68 μ_B).

Finally, total-energy calculations have been performed to check the magnetic ordering and anisotropy for this optimized geometry, which included the effect of the vdW interactions. We found an isotropic exchange coupling constant of $J = 1.046$ meV and a magnetic anisotropy energy of $E_a = 0.35$ meV (for $U_{eff} = 4$ eV). These values are in a good agreement with those obtained without taking vdW forces into account, see Table S2. Thus, at this level of theoretical treatment (that includes the vdW corrections) Fe-DCA/Au(111) also shows strong ferromagnetic exchange coupling and robust out-of-plane magnetic anisotropy.

Some of the results calculated with VASP were verified against those obtained using the GREEN code [16, 17] and its interface to the SIESTA DFT-pseudopotential package [18] within the GGA+U approach and a good agreement was found.

- Monte Carlo modelling.

Given the strong uniaxial single-ion anisotropy observed experimentally, we estimate the magnetization as a function of temperature using a basic honeycomb Ising Monte Carlo model essentially as implemented in Ref. 19

$$H = -\frac{J^{\mathrm{MC}}}{2} \sum_{i,j} S_i S_j. \quad \text{(S2)}$$

Using $J^{\mathrm{MC}} = 1.98$ meV and the experimental applied field (0.1T and 0.05T, respectively) the curves represented in Fig. 2e and Fig. S5e are obtained. Note that this $J^{\mathrm{MC}} = 1.98$ meV exchange constant is in reasonable agreement with the one obtained by DFT (see Table S2).

- Semi-empirical Multiplet Calculations.

First, we have performed semi-empirical multiplet calculations for the core-level spectra of a Fe($3d^6$) ion in C_{3v} point group using the Quanty software [20, 21] based on our XAS and XMCD dataset with the Crispy interface [22]. In this way we check the large experimental value of the spin moment. The calculated spectra shown in Fig. S9 take into consideration the same experimental conditions we used for our XAS and XMCD dataset, i.e., $T = 2$K, $H = 6$T, two different incidence angles (normal, $\phi = 0°$, and grazing incidence, $\phi = 70°$) and the magnetic field always parallel to the incoming X-ray beam.

The XAS and XMCD calculations that correspond to the case of Fe(II) are displayed on Fig. S9α, β and include spin-orbit coupling, crystal field (CF) effects, and a slight reduction of the Slater integrals. Simulations were performed with Fe(II) in C_{3v} symmetry, with the strength of the CF described by the empirical parameters $10Dq = 0.75$ eV, $Dt = -0.26$, $Ds = 0.015$ for Fe2$^+$. To account for the experimental broadening, these calculations were at the end convoluted by a Lorentzian of width Γ= 0.46 eV for the $L_{2,3}$ edges that introduces an intrinsic core-hole lifetime broadening and a Gaussian of σ = 0.3 eV to account for the instrumental broadening.

We obtain a very good agreement with the experimental curves of Fig. S9a,b and, especially when considering that the latter have been obtained by adjustment of the crystal field parameters

with just 3 variables. This means that we did not change any of the charge-transfer parameters introduced by default for the nominal Fe(II) spectrum, other than a slight reduction to 0.7 (from the default 0.8) of the Slater integral Fk. The analysis given by Quanty of the initial Hamiltonian gives $< S_z >= -1.98$, and $< L_z >= -0.97$, in excellent agreement with the many-body spin Hamiltonian with point charge crystal field calculation described just below.

- Multiplet calculations within a many-body Hamiltonian approach.

Electronic multiplet calculations were done to study the magnetic excitation spectrum corresponding to a single Fe atom surrounded by its first six nearest neighbours in the optimized geometry of the Fe-DCA metal organic coordination network deposited on the Au(111) surface. These six neighbours are three N atoms and another three Au atoms. In these calculations, all correlations between electrons sat on the $d-$shell orbitals of the central Fe atom are included, while hoppings to other Fe orbitals or surface atoms are neglected. The Coulomb interaction between these electrons is analytically calculated under the assumption of hydrogenic wave functions [23], and it is parameterized in terms of a single parameter, the average on-site Coulomb matrix element $U \equiv V_{ii,ii}$ [24, 25]. Here, we have taken $U = 5.27$ eV (low energy excitations are barely dependent on U for $U \in [3-7]$ eV). The spin-orbit interaction and the Zeeman terms are also evaluated analytically, as in Refs. [24, 25], with a value of the single-particle spin-orbit coupling strength of 50.09 meV, corresponding to Fe^{2+} in gas phase [26].

The effect of the surrounding atoms is included through a crystal field produced by classical point charges. The corresponding crystal-field Hamiltonian depends on the charges $\{q_j\}$ and positions $\{\mathbf{r}_j\}$ of the neighboring atoms, together with the expectation values $\langle r^2 \rangle$ and $\langle r^4 \rangle$ of the radial coordinate.

The common approach is to take the charges and expectation values $\langle r^2 \rangle$ and $\langle r^4 \rangle$ as fitting parameters since the agreement between calculated and experimental spectra are in general quite poor [27]. This is mainly due to the overestimation of the convergence of crystal field potential series associated with the finite extent of the charges distributions, together with covalent bonding and screening effects [28]. The main advantage of using a point-charge model (PCM) is that it properly reproduces the point symmetry of the magnetic atom. Additionally, the number of parameters can be greatly reduced using physical and chemical constraints [28] compared to alternative solutions based on pure phenomenological parametrizations as the Stevens' operators [26].

Hence, to avoid over-parametrization, we also used the tabulated values of $\langle r^2 \rangle = 1.393$ a.u. and $\langle r^4 \rangle = 4.496$ a.u. [26]. The values of the positions are taken from the DFT calculation of the Fe-DCA/Au(111) previously detailed. The values of the point charges are given in Table S3. To avoid spurious effects due to finite size simulations and artificial strains, we have symmetrized the positions to preserve the C_3 symmetry of the N and Au atoms around the Fe center (displacements always smaller than 5%). In addition, we screen the crystal-field by a factor 0.15 (the MAE depends only very weakly on this screening factor).

Figure S10a shows the low-energy magnetic excitations as a function of the spin-orbit coupling and applied out-of-plane magnetic field. As observed, the ten energy levels can be grouped into a lower quintuplet where the spin and orbital magnetic moments are aligned and an excited quintuplet with antialigned orbital and magnetic moments. Two main features are clearly observed: i) there is an easy axis anisotropy favoring an out-of-plane magnetic moment, and ii) there is a large unquenched orbital moment contribution which is maximum for the ground state, as also evinced by the spin and orbital moments displayed in Figure S10b and c. Although the actual values of (S_z, L_z) can display significant variations with the ratio $\langle r^2 \rangle / \langle r^4 \rangle$, the previously mentioned properties i) and ii) remain robust for sensible values of this ratio.

In essence, we obtain that the ground state multiplet corresponds to maximum $S_z = 1.97$ and $L_z = 1.05$ values of the spin and orbital momentum perpendicular to the surface, which is practically identical to the semi-empirical Multiplet calculations ($< S_z >= -1.98$, and $< L_z >= -0.97$). Moreover, the first excited state, which defines the magnetic anisotropy energy, is ~ 8.5 meV higher in energy. This is an order of magnitude larger than the DFT calculated value ($E_a = 0.6$ eV) and is expected to be closer to the experimental case.

S3. XMCD SUM RULES

A XMCD spectrum is the difference between two XAS spectra obtained with opposite circular polarizations (whose helicity is typically parallel or antiparallel to the applied magnetic field). Typically, several spectra must be accumulated to obtain the desired statistics with acceptable signal-to-noise ratio.

Once a high quality XMCD spectrum has been acquired, the orbital and spin contributions to the total magnetic moment can be, in principle, separately obtained. The tools allowing this are the magnetooptical sum rules derived by P. Carra, B.T. Thole and G. van der Laan in the 90's [29, 30]. They can be expressed as:

$$\mu_L = -\frac{2}{3}\frac{A+B}{C}n_h \tag{S3}$$

$$\mu_S^{\text{eff}} = \frac{2B-A}{C}n_h \tag{S4}$$

where n_h is the number of holes in the final electron states band (the $3d$ band for the Fe $L_{2,3}$ absorption edges), A and B are the area enclosed under the L_3 and L_2 edges of the XMCD spectrum, respectively, and C is the area enclosed in both edges of the XAS spectrum once the excitations to the continuum have been removed. A, B and C are calculated by integrating XAS and XMCD spectra. To remove the contribution from transitions to the continuum, typically a double-step function is subtracted from the XAS spectrum. With such a minute sample as the 2D-MOCN we are dealing here, it is essential to treat consistently the whole series of data to obtain a robust result.

The obtained parameter in equation S4 is $\mu_S^{\text{eff}} = \mu_S - \frac{7}{2}\mu_T$, where μ_S is the spin moment and μ_T the magnetic dipole moment. If the studied magnetic moment rotates with the magnetic field in an angle dependent experiment, the angle dependence of the orbital and effective spin magnetic moments is respectively given by [31]:

$$\mu_L(\varphi) = \mu_L^z \cos^2(\varphi) + \mu_L^{xy} \sin^2(\varphi) \tag{S5}$$

$$\mu_S^{\text{eff}}(\varphi) = \mu_S - 7[\mu_T^z \cos^2(\varphi) + \mu_T^{xy} \sin^2(\varphi)] \tag{S6}$$

where $\mu_S = -2\langle S_z\rangle\mu_{\text{B}}/\hbar$ is isotropic, but both the orbital moment $\mu_L(\varphi) = -\langle L_z^{\varphi}\rangle\mu_{\text{B}}/\hbar$ and the dipole magnetic moment of the spin density distribution $\mu_T(\varphi) = \langle T_z^{\varphi}\rangle\mu_{\text{B}}/\hbar$ are intrinsically anisotropic. Because the dipolar tensor is traceless, and therefore $\mu_T^z + 2\mu_T^{xy} = 0$, only four moment components in Eqs. S5 and S6 are independent. A particular case worth mentioning occurs at the so-called 'magic angle' of incidence ($\varphi^m = 54.7°$), where $2\cos^2(\varphi^m) = \sin^2(\varphi^m)$ so the dipolar term is canceled in Eq. S6, yielding a direct measure of μ_S [32].

As an example of this procedure we have fitted the orbital, effective spin and total moment of

the sample formed by Fe clusters on Au(111) to Eqs. S5 and S6, shown in Fig. S4c with the same color as the symbols. The 'magic angle' measurement allows to determine $\mu_S = 2.85 \pm 0.15\mu_{\mathrm{B}}$, and thus $\mu_T^z = 0.06 \pm 0.02\mu_{\mathrm{B}}$, while $\mu_L^z = 0.60 \pm 0.05\mu_{\mathrm{B}}$. However, the dependence of the Fe-DCA/Au(111) 2D-MOCN is completely different exhibiting a $\cos(\varphi)$ dependence (dashed lines in Fig. S4c). Clearly the two systems obey different angular behavior due to the large differences on the anisotropy of the orbital moment which in the case of Fe-DCA/Au(111) anchors the spin to the easy OOP axis through spin-orbit interaction.

S4. REDOX STATES DETERMINED BY XPS

We carried out XPS experiments to determine the redox state of the 2D-MOCN by measuring five representative samples: the Fe-DCA network, the DCA islands (as Fig. S1), Fe as nanodots nucleating at the herringbone elbows (as Fig. S2a), a high coverage of Fe and the pristine Au(111) substrate. For each, we consecutively acquired the core levels (CLs) of Fe2p, N1s and C1s and the O1s to check for undesired oxidation of the samples. The resulting XPS spectra for the important CLs are shown in Fig. S7, where we obtain the following results:

- The shown spectra are within the detection limit of the XPS instrument (very noisy signals) due to the minute amounts of Fe (less than 5% of a ML) and DCA.

- No oxidation was observed in the datasets based on the absence of an O1s peak in the vicinity of 530 eV.

- For the Fe2p signal, the largest contribution at the Fe2$p_{3/2}$ position corresponds to the Au(111) substrate that presents a weak Auger signal at that energy.

- The metallic state (Fe 0) lineshape and energy position was obtained measuring a high Fe coverage spectrum (Metallic Fe) of roughly 2 ML (707.7 eV for the Fe2$p_{3/2}$).

- For the Fe-DCA network sample (Network) the Fe2p is clearly from a different valence state compared to the metallic one previously mentioned. The average Fe2$p_{3/2}$ signal in the Network is estimated to be at $E_B \sim$ 709.3 eV, which agrees with an Fe 2+ oxidation state, in agreement with the XAS spectra (Fig. 2a).

- For the same amount of Fe as the network (Fe $< 5\%$) – but without molecules – forming

nanodots at the herringbone elbows, the corresponding lineshape is similar to the metallic state (Fe 0 state).

- The C1s lineshape shows two well defined components when the Fe atoms and molecules form the network (at 285.0 eV and 286.2 eV), which we attribute to the difference between the anthracene backbone carbon atoms and the carbon atoms binding to the N atoms. In the absence of metal coordination, the aggregated islands of DCA feature a dominant peak at 284.7 eV for this C1s component.

- Concerning the N1s signal, we observe an apparent shift of $\sim$ 1.0 eV to higher binding energies upon network formation, as compared to the molecules forming islands (400.1 eV vs 399.1 eV).

From these results we can conclude that the Fe centers coordinating the Fe-DCA network are in a 2^+ state, in agreement with the XAS/XMCD and theory. Moreover, the shift of the N1s CL to higher binding energies when forming the network compared to when forming islands without metal coordination agrees with the related network of Cu-DCA/Cu(111) [33]. As the cyano end-groups must deplete the electronic charge of the metal centers when forming the organometallic bonds [34], the 2^+ state of the Fe centers requires that 4 electrons are shared by the three surrounding molecules (stoichiometry is 2 Fe atoms to 3 DCA per unit cell). Interestingly, the shift to higher binding energies of the N1s and C1s can only be interpreted as a charge transfer from the conjugated network to the Au(111) substrate. Such electronic exchange should yield an upward shift of the DCA LUMO, which we confirmed by our DFT calculations in Fig. S11.

S5. ROLE OF THE AU(111) SUBSTRATE IN THE MAGNETIC PROPERTIES OF THE FE-DCA MOCN

To understand the role of the Au(111) substrate in the observed magnetic properties of Fe-DCA/Au(111), we consider the four following systems: the full Fe-DCA/(Au111), the planar free-standing (FS) Fe-DCA, the artificial Fe-honeycomb*/Au(111), and the optimized Fe-honeycomb/Au(111). Note the use of the asterisk (*) to distinguish between the two Fe-honeycomb-on-Au(111) systems, the differences between which are discussed in detail below. With these four cases, we can systematically correlate the crystal structures with their magnetism.

Tables S4 and S5 below summarize the magnetic characteristics of the four systems under consideration. The Fe-DCA/Au(111) is the "full" system that models the experimental 2D-MOCN. In this case, the Fe centers show local magnetic moments of 3.68 μ_B. The corresponding occupations of the $3d$ manifold, as well as the magnetic moments induced in the DCA molecules and on the neighboring Au atoms are indicated in the tables.

We now compare the full Fe-DCA/Au(111) to the planar free-standing Fe-DCA monolayer. The latter was fully optimized, i.e., the lattice parameter and atomic coordinates correspond to the energy minimum. We find an order of magnitude stronger FM exchange coupling strength compared to the Fe-DCA/Au(111) (Table S5), as well as different $3d$ occupations, especially those of the minority $3d_{z^2}$ and $3d_{x^2-y^2}$ orbitals (Table S4). These changes in occupations result in a somewhat smaller spin magnetic moments at the Fe centers (3.58 μ_B). Nevertheless, a significantly larger spin polarization appears to be induced in the DCA ligands compared to the Fe-DCA/Au(111) case (Table S5). The larger induced moment in DCA of the planar FS Fe-DCA correlates with the shorter Fe-N bond length as compared to Fe-DCA/Au(111), which is also consistent with the stronger exchange coupling between the Fe centers. Incidentally, the spin moment of the DCA molecules is opposite in sign to that of Fe, revealing AFM coupling between the Fe centers and DCA ligands (i.e., such kind of ferrimagnetism takes place in both Fe-DCA/Au(111) and planar FS Fe-DCA). These results show that the origin of the magnetic property of the Fe-DCA/Au(111) system lies in Fe-DCA network. Nevertheless, the Au(111) substrate introduces differences between these two systems in the form of network geometry modification, charge transfer and electronic hybridization between the network and the underlying Au(111) surface. The charge transfer can be observed by comparing the electronic band structure near the Fermi level of both systems, i. e., Fe-DCA/Au(111) and FS Fe-DCA in Fig. S11.

Next, we consider the two auxiliary systems, the non-optimized Fe-honeycomb*/Au(111) and the optimized Fe-honeycomb/Au(111), to justify the validity of our estimation of the strength of the exchange coupling J_{substr}, mediated by the Au(111) surface.

The Fe-honeycomb*/Au(111) is an artificial system, derived from the fully-optimized Fe-DCA/Au(111) by removing the DCA molecules while leaving the Fe atoms exactly at the same positions. Thus, it forms a 2D Fe honeycomb with the adsorption height of about 2.5 Å above the Au surface. It is important to emphasize that this adsorption height is larger than the equilibrium one,

corresponding to the Fe-honeycomb/Au(111) case. Crucially, we purposely fix the Fe-$3d$-manifold occupations in Fe-honeycomb*/Au(111) to those of Fe in the Fe-DCA/Au(111) system. This is done using the occupation matrix control (OMC) method [10], which is used to pre-converge the charge density using the occupation constraint to then perform a standard DFT calculation without constraints. In this way, we obtain the magnetic configuration of Fe-honeycomb*/Au(111) with $3d$ occupations of the Fe atoms that differ at most by $\sim$0.03 (i.e., $\sim$3 %) from those in the full Fe-DCA/Au(111) system (Table S4). As a result, the magnetic moment contributed by the Fe-$3d$ states (not to be confused with the local magnetic moment m given in Tables S4 and S5, which contains contributions of all Fe valence states) differs only by $\sim$0.057 μ_B between these two systems. The difference in the Fe local magnetic moments m of about 0.2 μ_B between Fe-honeycomb*/Au(111) and Fe-DCA/Au(111) comes from the magnetization of the Fe-$4s$ states (Table S4). The occupation of the Fe-$4s$ states (and hence their magnetization) has not been controlled within OMC. Thus, (i) the Fe-$3d$ occupations are practically the same in both Fe-honeycomb*/Au(111) and Fe-DCA/Au(111), and (ii) the lateral separation between the Fe atoms as well as their adsorption height above Au(111) turn out to be exactly the same. The points (i) and (ii) give grounds for the use of this tailored Fe-honeycomb*/Au(111) system to estimate the scale of the strength of J_{substr}, which is the exchange coupling mediated by the Au(111) surface in the full Fe-DCA/Au(111) system.

The exchange coupling between Fe spins in this model auxiliary system Fe-honeycomb*/Au(111) is more than an order of magnitude weaker than in Fe-DCA/Au(111), see Table S5. This strongly implies that the Fe-Fe superexchange via DCAs dominates over the RKKY-type exchange through Au(111). However, it should be emphasized that the calculation for Fe-honeycomb*/Au(111) can only provide an order of magnitude estimate because the Fe atoms in Fe-honeycomb*/Au(111) and Fe-DCA/Au(111) are not in an identical state.

Having compared the strengths of the Fe-Fe exchange via DCAs and via Au(111), one may also compare the estimated J_{substr} to the strongest possible Fe-Fe exchange in this Fe-honeycomb-on-Au(111) for the fixed Fe-Fe lateral separation. To do this, we allow the Fe atoms of the Fe-honeycomb*/Au(111) to find their equilibrium adsorption height, while keeping their xy coordinates fixed. We will refer to the resulting system as Fe-honeycomb/Au(111) (without asterisk). As a result of such a structural relaxation, the Fe atoms end up being significantly closer to the substrate (adsorption height of $\sim$1.82 Å in comparison to $\sim$2.5 Å before relaxation in the Fe-honeycomb*/Au(111)

system), resulting in a shorter Fe-Au bond length by 12 %. A very important conclusion we draw here is that the Fe-Au hybridization in Fe-honeycomb*/Au(111) [hence, in Fe-DCA/Au(111)] is rather weak.

Furthermore, for the Fe-honeycomb/Au(111) we do not fix the occupation of the Fe-3d orbitals at all, but instead perform a self-consistent optimization of the electronic degrees of freedom to find the optimal occupancies without constraints. This results in a different Fe-3d occupation numbers and lower spin moment as compared to Fe-honeycomb*/Au(111). Notably, the J_{substr} absolute value in this case turns out to be an order magnitude larger than in Fe-honeycomb*/Au(111) system (see Table S5). However, even under this strongest interaction case, J_{substr} is still several times weaker than in the full Fe-DCA/Au(111) system. Note that $J_{substr} < 0$ for Fe-honeycomb/Au(111), i.e. the coupling between the Fe centers is antiferromagnetic. It should be said that in both Fe-honeycomb/Au(111) and Fe-DCA/Au(111) cases, very small magnetic moments are induced by the Fe atoms at the three Au atoms nearest neighbors (two and three orders of magnitude smaller than Fe's moment).

Summarizing the above analysis of structure and magnetism of the Fe-DCA/Au(111), planar FS Fe-DCA, Fe-honeycomb*/Au(111), and Fe-honeycomb/Au(111) systems, we conclude that the role of the Au(111) surface in determining the exchange coupling between Fe spins in Fe-DCA/Au(111) via an RKKY-type channel is quite limited. Indeed, the coupling through this channel is an order of magnitude weaker than the superexchange mediated by the DCA molecules. This result is consistent with the estimated value of the exchange coupling constant $J = 0.1$ meV between Mn and Fe atoms in a long-range magnetic 2D supramolecular Kondo lattice of FeFPc and MnPc grown on Au(111) [35].

However, we do not claim that the Au(111) does not play any role in the magnetism of this MOCN. Indeed, after formation on the substrate, the network becomes distorted from its planar geometry inducing changes in the magnetic state of the Fe atoms (3d-states occupation and corresponding spin moment), as well as a weakening of spin polarization of the DCA ligands. In this respect, we must emphasize that the N-Fe bond length increases by about 8 % from the free-standing Fe-DCA monolayer case to the Fe-DCA/Au(111) system. In this way, the role of Au(111) is not negligible in determining the exchange coupling between Fe centers, but it is rather indirect as it introduces shifts in the Fe and DCA levels due to a charge transfer and hybridization, which translates into

an ”effective” screening of the exchange coupling between Fe centers across the DCA ligands.

S6. SUPPLEMENTARY TABLES

	μ_L^z (μ_B)	μ_S^{eff} (μ_B)	μ_{tot}^z (μ_B)
Fe-DCA/Au(111)	1.88 ± 0.02	4.02 ± 0.04	5.90 ± 0.06
Fe/Au(111)	0.60 ± 0.05	2.85 ± 0.15	3.85 ± 0.20

TABLE S1. Orbital and spin magnetic moments of Fe in Fe-DCA/Au(111) and Fe/Au(111) obtained fitting the points in Figs. 2 and S4 and following Eqs. S5 and S6.

U_{eff} (eV)	vdW	m (μ_B)	S	$\Delta_{A/F}$ (meV per Fe pair)	J (meV)	E_a (meV per Fe atom)
4	No	3.68	2	31.7	1.321	0.55
4	Yes	3.65	2	25.1	1.046	0.35
5	No	3.75	2	25.9	1.079	0.55
6	No	3.80	2	17.4	0.725	0.66

TABLE S2. Calculated magnetic parameters of Fe-DCA/Au(111) for three different Hubbard U_{eff} values. For $U_{eff} = 4$ eV the results obtained with and without taking van der Waals (vdW) corrections into account are given. m (μ_B) – local magnetic moment on the Fe atom, S – spin, $\Delta_{A/F} = E_{AFM} - E_{FM}$ (meV per Fe pair), J (meV) – nearest neighbor Heisenberg exchange coupling parameter, E_a (meV per Fe atom) – magnetic anisotropy energy.

Atom type	Charge (e)	Position		
		x(Å)	y(Å)	z(Å)
N	-1.24	-1.8628	0.0093	0.9698
N	-1.24	0.9234	-1.6179	0.9698
N	-1.24	0.9394	1.6086	0.9698
Au	0.12	1.4425	0.8425	-2.3286
Au	0.12	-1.4509	0.8280	-2.3286
Au	0.12	0.0084	-1.6705	-2.3286

TABLE S3. Atom types, charges $\{q_j\}$ (in units of the electron charge e) and positions of the first three N and Au neighbors of the Fe atom used in the multiplet calculations within a many-body spin Hamiltonian approach. The Fe is located at the origin $\mathbf{r}_{\mathrm{Fe}} = \mathbf{0}$.

		Spin-up Fe-3d occupations					Spin-down Fe-3d occupations				
	m (μ_B/Fe)	d_{xy}	d_{yz}	d_{z^2}	d_{xz}	$d_{x^2-y^2}$	d_{xy}	d_{yz}	d_{z^2}	d_{xz}	$d_{x^2-y^2}$
Fe-DCA/Au(111)	3.680	0.9594	0.9653	0.9681	0.9927	0.9950	0.0201	0.0288	0.0645	0.0730	0.9449
Planar FS Fe-DCA	3.576	0.9790	0.9545	0.9495	0.9495	0.9790	0.0998	0.0930	0.9170	0.0930	0.0998
Fe-honeycomb*/Au(111)	3.888	0.9554	0.9580	0.9595	0.9596	0.9611	0.0050	0.0236	0.0576	0.0648	0.9501
Fe-honeycomb/Au(111)	3.220	0.9371	0.9381	0.9550	0.9551	0.9555	0.0122	0.0123	0.0180	0.7668	0.7802

TABLE S4. Local magnetic moments on the Fe atoms, m (μ_B/Fe), as well as the occupations of the Fe-3d manifold for the four following systems: full Fe-DCA/(Au111), planar free-standing (FS) Fe-DCA, artificial Fe-honeycomb*/Au(111), and optimized Fe-honeycomb/Au(111). Note that the Fe-3d occupations are very similar in the Fe-DCA/Au(111) and Fe-honeycomb*/Au(111) systems, which is achieved using the occupation matrix control (OMC) method. Given practically equal 3d occupations, the difference in the local magnetic moments between these two cases comes mainly from the Fe-4s states contribution, which is about 0.2 μ_B in Fe-honeycomb*/Au(111), but roughly an order of magnitude smaller in Fe-DCA/Au(111). The results were obtained for the Hubbard parameter $U_{eff} = 4$ eV.

	J (meV)	m (μ_B/Fe)	m_{DCA} (μ_B/DCA unit)	m_{Au} (μ_B/Au)	$d_{\text{Fe-N}}$ (Å)	$d_{\text{Fe-Au}}$ (Å)	$d_{\text{C-N}}$ (Å)
Fe-DCA/Au(111)	+1.321 (FM)	3.680	-0.096	+0.01	2.10	2.86	1.18
Fe-DCA/Au(111) [vdW]	+1.046 (FM)	3.650	-0.092	+0.01	2.06	2.79	1.18
Planar FS Fe-DCA	+10.663 (FM)	3.576	-0.236	-	1.94	-	1.19
Fe-honeycomb*/Au(111)	+0.038 (FM)	3.888	-	+0.018	-	2.86	-
Fe-honeycomb/Au(111)	-0.340 (AFM)	3.220	-	|0.002|	-	2.54	-

TABLE S5. Isotropic Heisenberg exchange coupling constants J (meV), local magnetic moments on the Fe atoms, m (μ_B/Fe), as well as induced magnetic moments in DCA (where applicable) and on the Au atoms that are Fe's nearest neighbors (where applicable) for the same systems as in Table S4 as well as Fe-DCA/Au(111) optimized taking van der Waals corrections into account (marked with [vdW]). Also shown are the $d_{\text{Fe-N}}$, shortest $d_{\text{Fe-Au}}$, and the cyano group $d_{\text{C-N}}$ distances. The absolute value of m_{Au} is given for Fe-honeycomb/Au(111) because this system shows antiferromagnetic coupling and, hence, the induced moment sign depends on the Fe local moment direction. Note that when calculating $J = \Delta_{\text{A/F}}/6S^2$ for Fe-honeycomb/Au(111) we take S = 3/2, since its local magnetic moment is close to 3 μ_B, unlike in other three systems where it is closer to 4 μ_B (S = 2). The results were obtained for the Hubbard parameter $U_{eff} = 4$ eV.

S7. SUPPLEMENTARY FIGURES

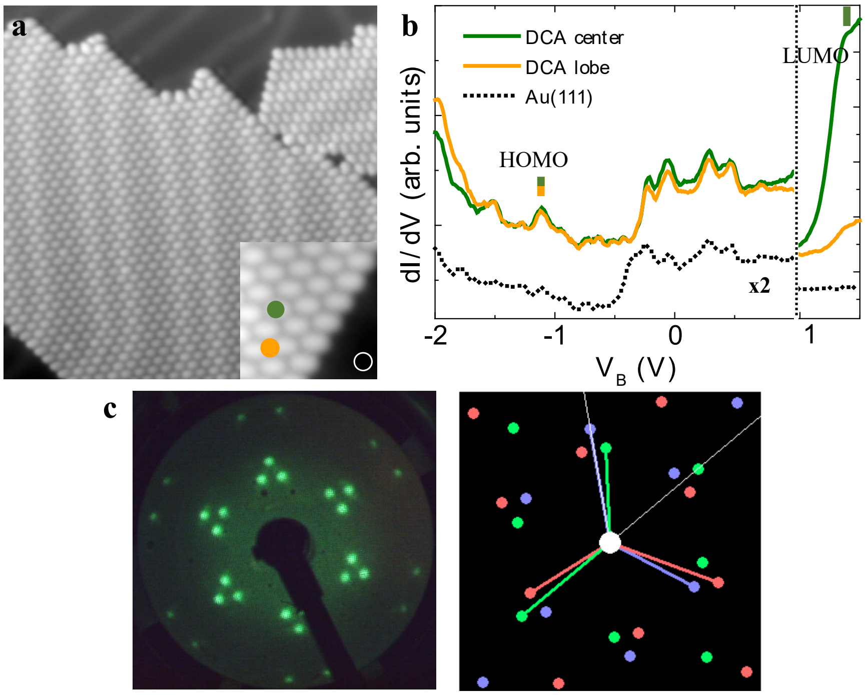

FIG. S1. Atomic and electronic characterization of DCA islands on Au(111). a) STM overview showing that the molecules aggregate into compact islands without metal coordination. Note that the herringbone reconstruction is unaffected below the molecular self-assembly. b) STS acquired at the indicated positions of the inset image in a, with different intensity scaling above and below 1 V. c) LEED characterization of the sub-monolayer DCA coverage on Au(111) without metal coordination and the corresponding simulated pattern. The arrangement corresponds to a rectangular oblique phase with vectors $a_1 = 1.18$ nm and $a_2 = 0.93$ nm and angle 50.8° [matrix notation: $\left(\begin{smallmatrix} 4.1 & 0 \\ 3.5 & 2.9 \end{smallmatrix}\right)$] that contains three different domains. $E_{beam} = 19$ eV, T_s = RT. STM details: a) 30×30 nm^2, I_t = 100 pA, $V_B = -1$ V. Inset: 5×5 nm^2, I_t = 100 pA, $V_B = -1$ V. b) STS setpoint: $I_t = 100$ pA, $V_B = -1$ V; $V_{rms} = 9.6$ mV, $f_{osc} = 817$ Hz.

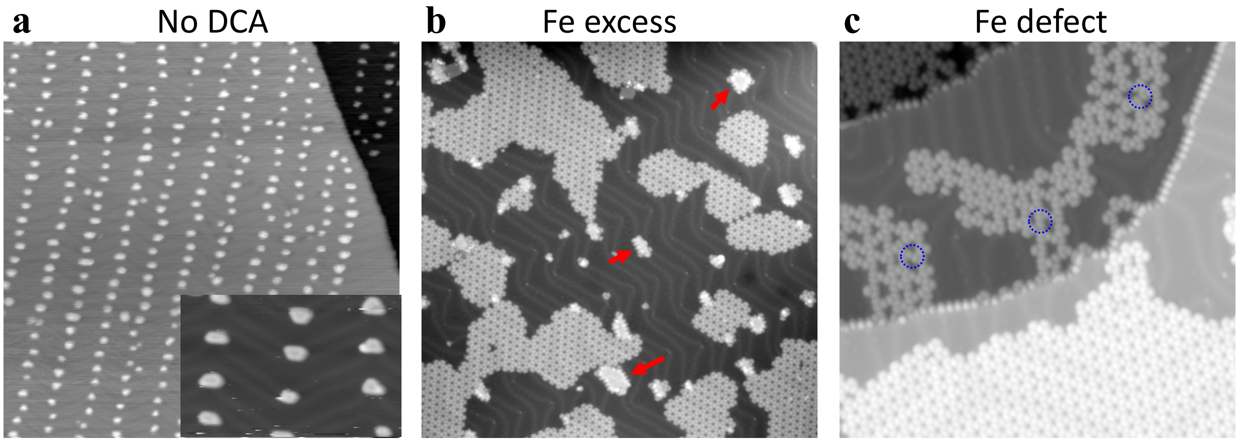

FIG. S2. Structural evolution modifying the Fe-DCA stoichiometry. a) Direct deposition of Fe on Au(111) without DCA recorded at RT in the same stoichiometry as a full monolayer of the network. The atoms cluster into small triangular islands at the elbows of the herringbone reconstruction. b) Upon slight excess of Fe atoms compared to the DCA molecules, the network still dominantes yielding the highly regular structure with practically no internal defects. The Fe excess clusters into islands externally decorated by DCA as if they were step edges (indicated by red arrows). c) Lack of Fe on the surface results in irregular structures with enlarged pores originating from metal-uncoordinated cyanos. These "metal-free" cyanos are electrostatically bonded by dipole-dipole interactions to neighbouring DCAs (cf. blue dotted circles) so that the metal-organic trimers separate, thereby enlarging laterally the pores. STM details: a) 200×200 nm^2, -0.003 V, 100 pA, inset: 39×25 nm^2, 1.0 V, 10 pA; b) 100×100 nm^2, -1.0 V, 100 pA; c) 50×50 nm^2, -1.0 V, 100 pA.

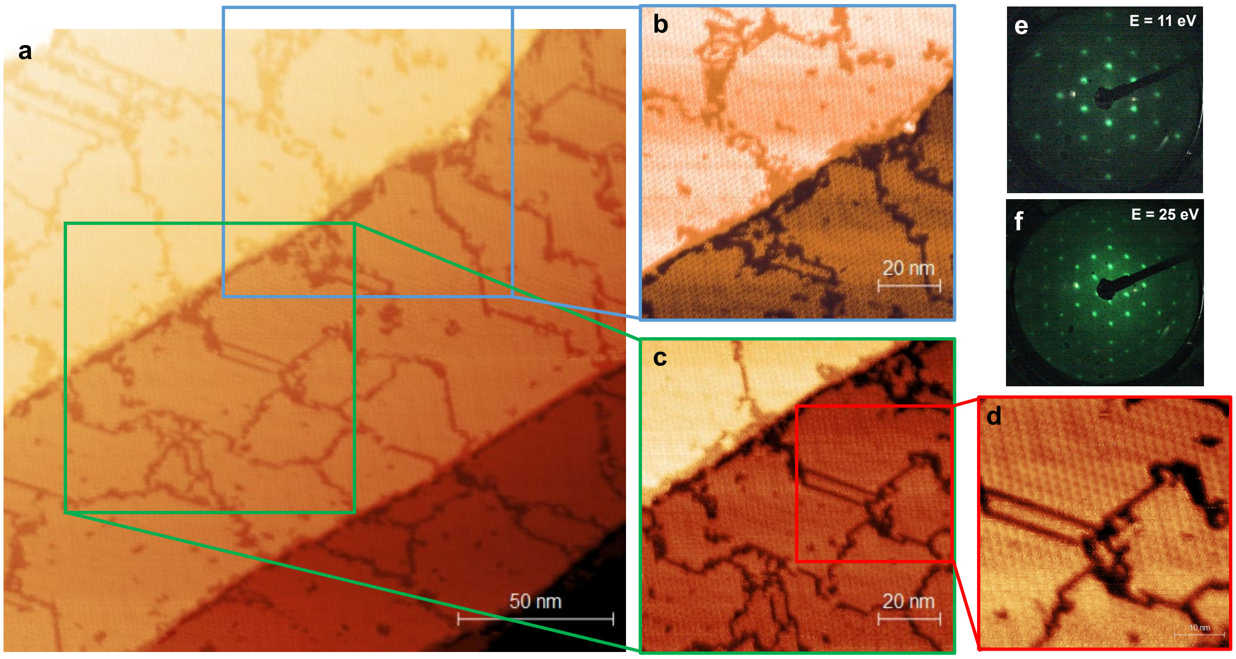

FIG. S3. Topography and LEED pattern of the sample measured by XMCD in Fig. 2 of the main manuscript acquired after the beam exposure. The STM overview a) and close ups b) - d) were acquired at room temperature after the XMCD acquisition. Note that no trace of Fe clusters can be detected on these STM images, exclusively showing the Fe-DCA 2D-MOCN. e) and f) display the room temperature LEED pattern at two different energies of the freshly prepared sample before being transferred to the XMCD setup. STM details: a) V= 0.8V, I = 400 pA, size $200 \times 200 nm^2$; b) V= 0.8V, I = 400 pA, size $100 \times 100 nm^2$; c) V= 0.5V, I = 400 pA, size $100 \times 100 nm^2$; d) V= 0.5V, I = 400 pA, size $50 \times 50 nm^2$.

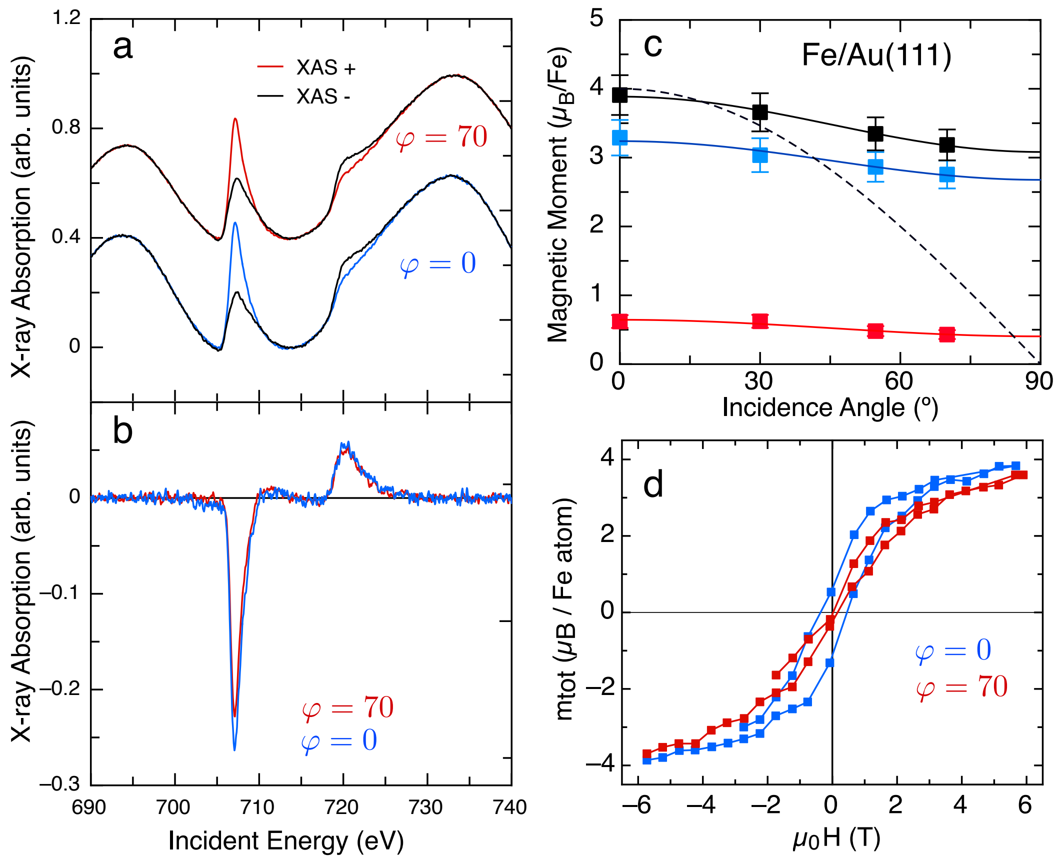

FIG. S4. Magnetic experiments obtained for the Fe/Au(111) system at ≈ 3 K: XAS (a) and XMCD (b) spectra obtained at normal (0°) and grazing (70°) incidence, (c) angular dependence of the orbital and spin effective moments, and (d) the hysteresis cycles at normal (0°) and grazing (70°) incidence. The amount of Fe on the surface was controlled to be identical to the experimental case of Fe-DCA/Au(111) network shown in Fig. 2 of the main manuscript. Several aspects must be underlined when comparing this graphs with the 2D-MOCN system: First, XAS and XMCD smooth lineshapes in (a) and (b) show a metallic character, very different to the ones shown in Fig. 2a. Second, the anisotropy of this Fe/Au(111), although being OOP as in Fe-DCA/Au(111), is much weaker. Third, the orbital moment in the Fe/Au(111) (red squares in (c)) is practically angle independent (the discontinuous line marks the scaled cosine relation $\mu_{Total}^{\varphi=0} \cdot \cos(\varphi)$ followed by the MOCN in Fig. 2c) and its magnitude is about 15% of the total moment. This is quite high for metallic Fe, but is probably due to the high surface to volume ratio in the formed clusters. However, in the 2D-MOCN its value is even higher (one third of the total magnetic moment) and clearly dominates the uniaxial character of the total moment (spin included). In contrast, in panel (c) the Fe clusters on Au(111) have slightly anisotropic magnetic moments (blue squares: effective spin, black ones: total moment). Finally, the coercive field of Fe on Au(111) is significantly lower than in the 2D-MOCN: it is just 0.5 T for normal incidence, and the grazing incidence curve displays a butterfly hysteresis loop that is probably related with slow relaxation of superparamagnetic units.

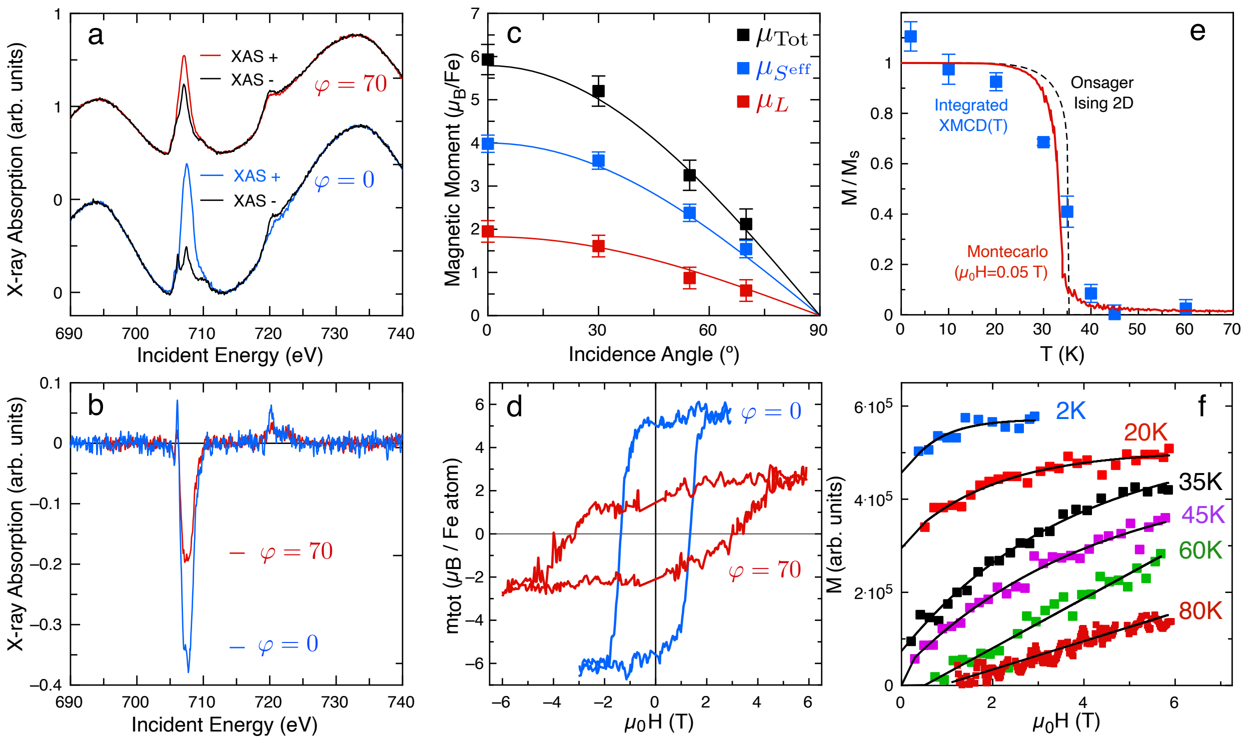

FIG. S5. First run measurements of the Fe-DCA network on Au(111) by XMCD. These measurements were acquired using a different Au(111) substrate and with one year difference to the ones shown in Fig. 2 of the main manuscript. Data reproducibility is unquestionable judging the match of the two results. XAS (a) and its corresponding XMCD (b) spectra acquired with circularly right (I^+) and left (I^-) polarized X-ray beam for normal (0°) and grazing (70°) incidence at the $L_{2,3}$ edges of Fe. (c) Angular dependence of the orbital (red), effective spin (blue) and total (black) magnetic moments obtained from the sum rules in the first experimental run. Note the reproducibility of the $\cos(\varphi)$ dependence and the normal incidence values $\mu_L^z \approx 2\ \mu_B$, $\mu_S^{\text{eff}_z} \approx 4\ \mu_B$, consistent with a Fe(II) d^6 high-spin (HS) configuration with $\langle L_z \rangle = 2$ and $\langle S_z \rangle = 2$. (d) Hysteresis loop measured at the L_3 edge of Fe at normal (blue) and grazing (red) incidence. The coercive fields of the open loops are slightly smaller than in the main manuscript ($\approx$ 1.5 T for the out-of-plane and $\approx$ 3.5 T for in-plane). We attribute these reduction to a slightly deffective preparation of the network on the surface on this run. (e) Plot of the temperature dependent area enclosed in XMCD L_3 edge from the spectra in Fig. S6 fitted to the Onsager analytical solution (discontinuous line) and to a Monte Carlo simulation (red line). (f) Magnetization isotherms at normal incidence ramping the field from 0 to 6 T. Black lines are guide to the eyes (curved and linear below and above T_C, respectively), as the noise level on these measurements does not allow to perform an Arrot plot representation nor a critical analysis. Note however, that remanence is evident at and below $T_C \approx 35$ K.

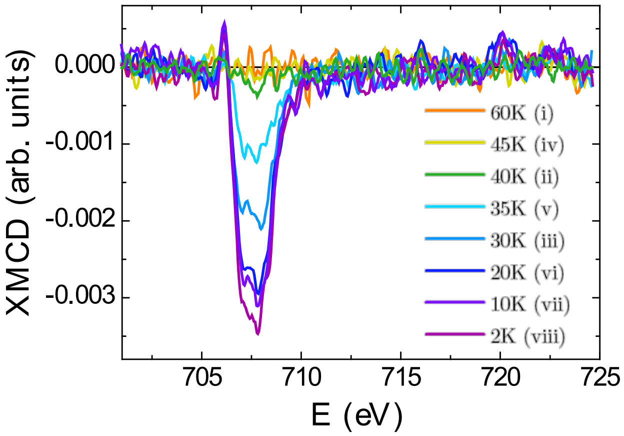

FIG. S6. Temperature dependence of the 2D-MOCN XMCD spectra at the $L_{2,3}$ edges of Fe on the first experimental run. These XMCD spectra were sequentially recorded at normal incidence ($\varphi = 0°$) with an external field of just H= 0.050 T. The roman numbers next to the temperatures indicate the measurement order: we started from RT and decreased the temperature until clear detection of the XMCD signal occurs at $T = 30$ K. Then, the system was warmed up to $T = 45$ K, where we confirmed the practical disappearance of the XMCD peaks. For a second time, we cooled down until reaching $T = 3$ K. The enclosed area of this peak was used to compose Fig. S4d, which is fully compatible within the experimental uncertainty with the experimental dataset of Fig. 2e (we stress that the magnetic field used here was just 50 mT). Moreover, the time sequence of these measurements provides further evidence of the temperature reversibility of this FM system and the absence of thermal hysteresis around $T_C \approx 35$ K.

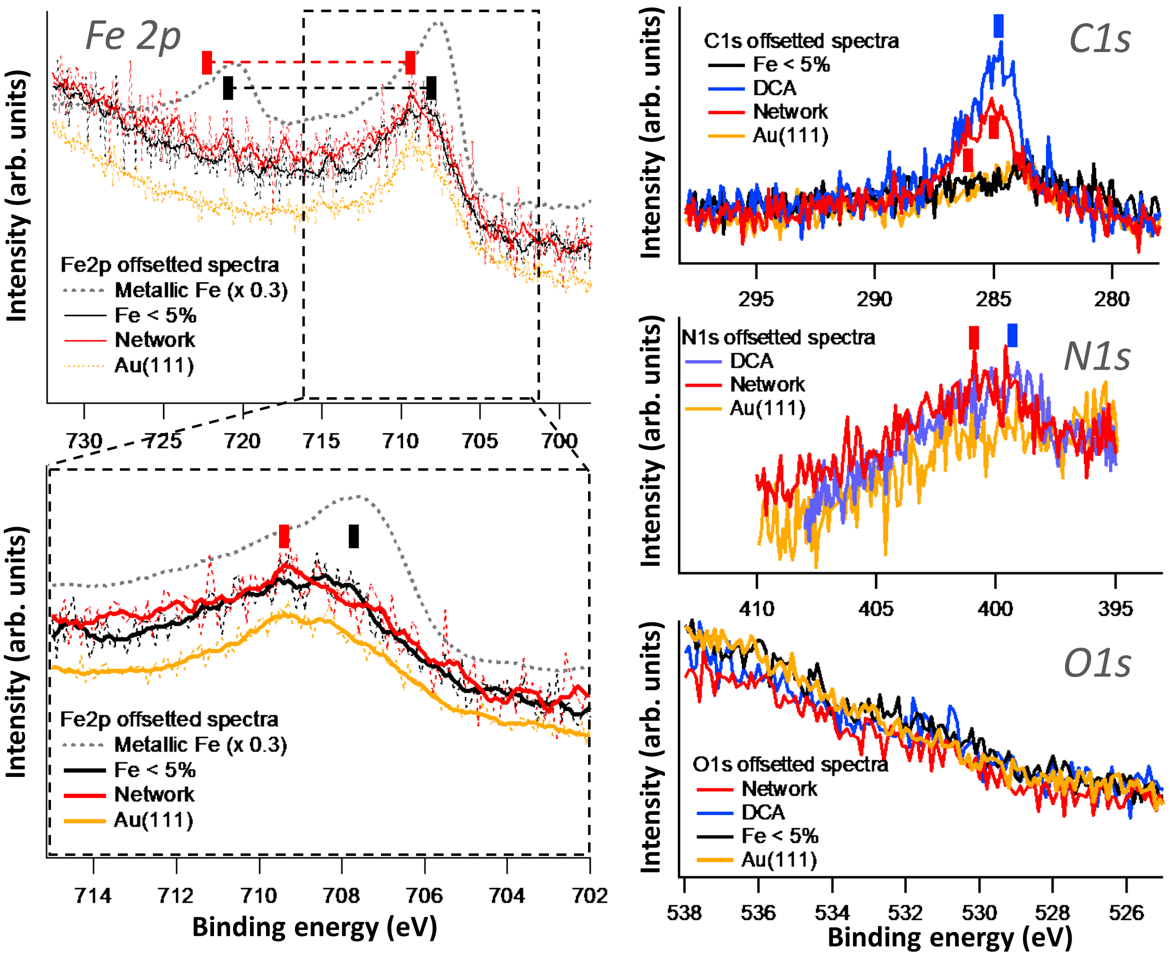

FIG. S7. XPS of the different core level acquired with a non-monochromatized Al K_α source for five different samples: Fe-DCA network (Network, red spectra), the DCA islands (DCA, blue spectra), Fe as nanodots nucleating at the herringbone elbows (Fe $< 5\%$, black spectra), a high coverage of Fe (Metallic Fe, gray dotted spectra) and the pristine substrate (Au(111), orange spectra). Despite the long acquisition times (above one hour per spectrum) these spectra were at the detection limit of our equipment (see Experimental methods section). In the left column the two components of the Fe2p core level is shown at the top, whereas at the bottom a close up on the Fe2$p_{3/2}$ peak is represented for visualization purposes. Due to the noisy condition of the spectra, a smoothing in an energy window of 1.1 eV is performed for the Fe2p (continuous lines) and the data are offset for clarity. For the C1s, N1s and O1s, the spectra are offset to coincide at the lowest binding energy side. The ticks in the panel mark the estimated peak position energies. Consult Section S4 for details.

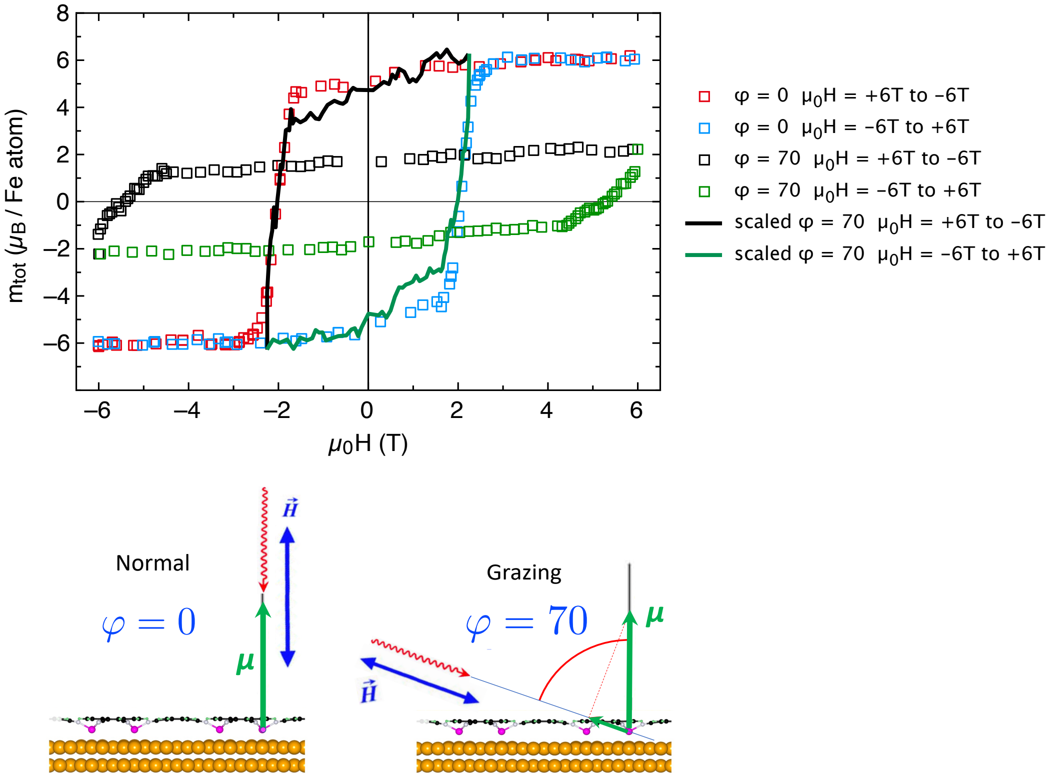

FIG. S8. Hysteresis loops (open symbols) obtained at the L_3 edge of Fe at normal ($\varphi = 0°$) and grazing ($\varphi = 70°$) incidence (same data as in Fig. 2d of the main paper). Here the 4 branches (two for each angle incidence) have been singled out with different colors, as indicated in the legend. This figure emphasizes that the $\varphi = 0°$ loop (red and blue squares) can be calculated in very good approximation from the $\varphi = 70°$ curves (black and green squares) assuming the OOP magnetic moment (green vector in the lower figures) is not affected by more than $\pm 2°$ by the 6T acting on the $\varphi = 70°$ geometry. As explained in the main text, the field along $\varphi = 0°$ when measuring at $\varphi = 70°$ is just the component of the applied field at $H_{0°} = H_{70°} \cos(70°)$ and correspondingly the magnetization is $M_{0°} = M_{70°}/\cos(70°)$. For instance, if we take the $\varphi = 70°$ dataset (green and black open squares) and calculate its projection it yields the green and black lines. Remarkably these lines practically reproduce the $\varphi = 0°$ experimental dataset (blue and red open squares). This evidences the intense uniaxial rigidity of the magnetic moments of Fe(II) in the Fe-DCA/Au(111) system, and the strongly hard magnetic character of the 2D-MOCN.

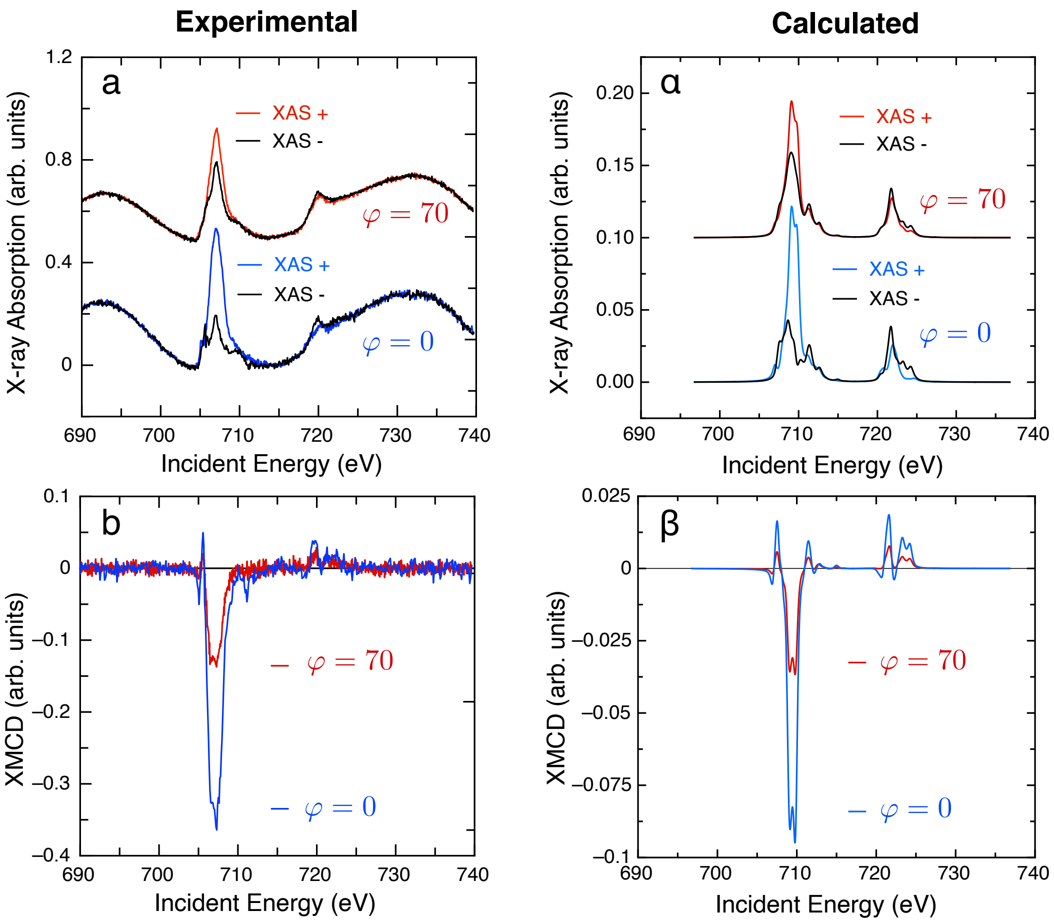

FIG. S9. Comparison between the XAS/XMCD lineshapes corresponding to the experimental (a,b) and multiplet calculated (α, β) Fe-DCA/Au(111) system. The left panels (experimental) are the ones from Fig. 2a,b, whereas the right ones have been semi-empirically generated with the Crispy/Quanty software [20–22] assuming a Fe 2^+ ($3d^6$) ion in C_{3v} point group. The good agreement between the two confidently provides a very strong theoretical support of the 2+ Fe(II) state in Fe-DCA/Au(111) network. Calculation details: The calculations include spin-orbit coupling, crystal field (CF) effects (described by the empirical parameters $10Dq = 0.75$ eV, $Dt = -0.26$, $Ds = 0.015$), and a slight reduction of the Slater integrals, which are convoluted with a Lorentzian of Γ = 0.46 eV to account for intrinsic core-hole lifetime broadening and by a Gaussian of σ = 0.3 eV to account for instrumental broadening.

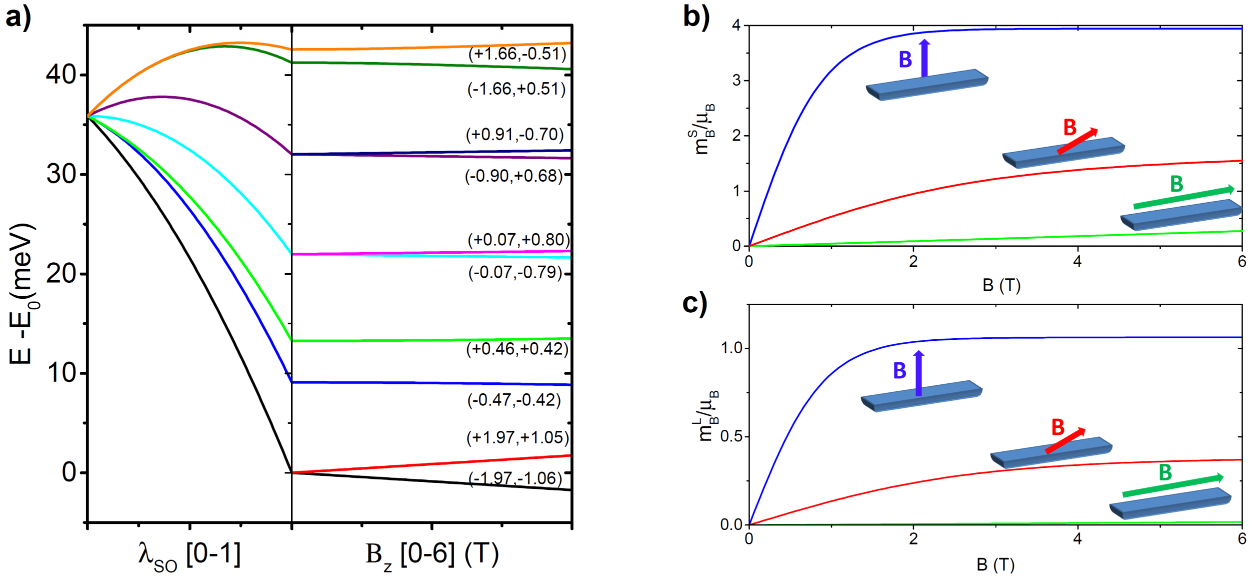

FIG. S10. (a, left side of the graph) Splitting of the lowest energy multiplet ($S = L = 2$) as one adiabatically connects the spin-orbit coupling up to the free ion value. (a, right side of the graph) Splitting of the energy levels with the out-of-plane magnetic field. The values in brackets close to the energy levels indicates the expectation values of S_z and L_z obtained at 6 T. (b) Spin and (c) orbital magnetic moments in units of the Bohr magneton calculated assuming thermal equilibrium at $T = 4$ K for a magnetic field applied out-of-plane (blue), grazing $\phi = 70°$ (red) and in-plane (green).

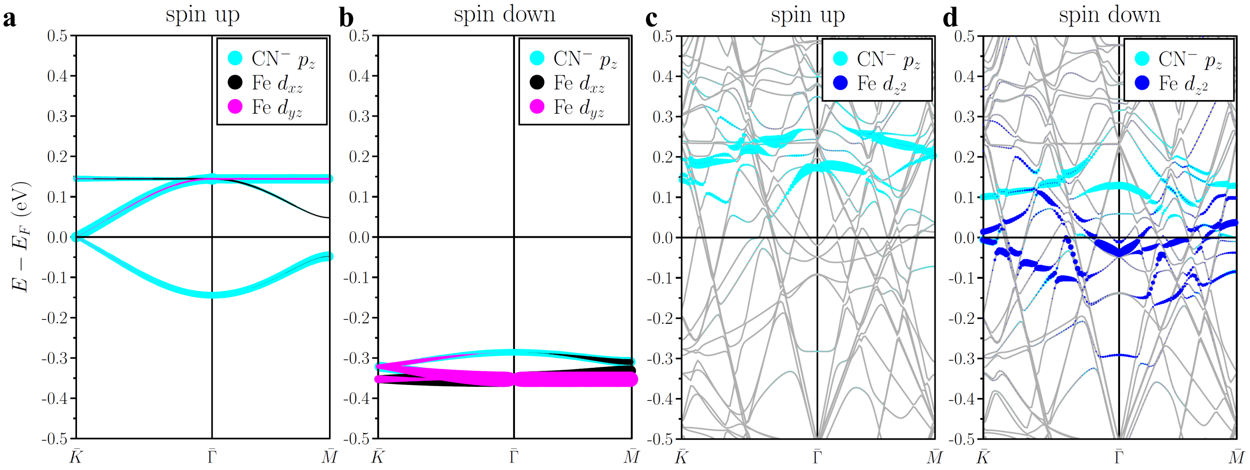

FIG. S11. Calculated low-energy band structures of the planar free-standing Fe-DCA (a,b) and full Fe-DCA/Au(111) system (c,d). Panels (a,c) and (b,d) correspond to the spin-up and spin-down channels, respectively. Color circles show projections onto the CN$^-$ p_z (cyan), Fe d_{xz} (black), d_{yz} (magenta) and d_{z^2} (blue) orbitals. Note the change of the orbital character of the Fe states near the Fermi level upon placing the Fe-DCA network on the substrate. A strong electronic hybridization between the organic ligands and the Fe centers can be seen in the spin-down channel. Grey lines in (c,d) correspond to the slab bands and are essentially the Au states. A clear up-shift of the Fe- and CN$^-$-derived bands towards the unoccupied states occurs in the presence of the substrate, indicating the charge transfer from the MOCN to the latter. The results were obtained for the Hubbard parameter U_{eff} =4 eV.

[1] L. Hernández-López, I. Piquero-Zulaica, C. A. Downing, M. Piantek, J. Fujii, D. Serrate, J. E. Ortega, F. Bartolomé, and J. Lobo-Checa, “Searching for kagome multi-bands and edge states in a predicted organic topological insulator,” *Nanoscale*, vol. 13, no. 10, pp. 5216–5223, 2021.

[2] A. Barla, J. Nicolás, D. Cocco, S. M. Valvidares, J. Herrero-Martín, P. Gargiani, J. Moldes, C. Ruget, E. Pellegrin, and S. Ferrer, “Design and performance of boreas, the beamline for resonant x-ray absorption and scattering experiments at the alba synchrotron light source,” *Journal of Synchrotron Radiation*, vol. 23, pp. 1507–1517, Nov 2016.

[3] P. E. Blöchl, “Projector augmented-wave method,” *Phys. Rev. B*, vol. 50, pp. 17953–17979, Dec 1994.

[4] G. Kresse and J. Furthmüller, “Efficient iterative schemes for ab initio total-energy calculations using a plane-wave basis set,” *Phys. Rev. B*, vol. 54, pp. 11169–11186, Oct 1996.

[5] G. Kresse and D. Joubert, “From ultrasoft pseudopotentials to the projector augmented-wave method,” *Phys. Rev. B*, vol. 59, pp. 1758–1775, Jan 1999.

[6] J. P. Perdew, K. Burke, and M. Ernzerhof, “Generalized gradient approximation made simple,” *Phys. Rev. Lett.*, vol. 77, pp. 3865–3868, Oct 1996.

[7] D. D. Koelling and B. N. Harmon, “A technique for relativistic spin-polarised calculations,” *J. Phys. C: Sol. St. Phys.*, vol. 10, no. 16, p. 3107, 1977.

[8] V. I. Anisimov, J. Zaanen, and O. K. Andersen, “Band theory and mott insulators: Hubbard U instead of stoner I,” *Phys. Rev. B*, vol. 44, pp. 943–954, 1991.

[9] S. L. Dudarev, G. A. Botton, S. Y. Savrasov, C. J. Humphreys, and A. P. Sutton, “Electron-energy-loss spectra and the structural stability of nickel oxide: An LSDA+U study,” *Phys. Rev. B*, vol. 57, pp. 1505–1509, Jan 1998.

[10] J. P. Allen and G. W. Watson, “Occupation matrix control of d-and f-electron localisations using dft+ u,” *Physical Chemistry Chemical Physics*, vol. 16, no. 39, pp. 21016–21031, 2014.

[11] W.-B. Zhang, Q. Qu, P. Zhu, and C.-H. Lam, “Robust intrinsic ferromagnetism and half semiconductivity in stable two-dimensional single-layer chromium trihalides,” *Journal of Materials Chemistry C*, vol. 3, no. 48, pp. 12457–12468, 2015.

[12] J. L. Lado and J. Fernández-Rossier, “On the origin of magnetic anisotropy in two dimensional cri_3,” *2D Materials*, vol. 4, p. 035002, jun 2017.

[13] C. Wäckerlin, A. Cahlík, J. Goikoetxea, O. Stesovych, D. Medvedeva, J. Redondo, M. Švec, B. Delley, M. Ondráček, A. Pinar, *et al.*, "The role of the magnetic anisotropy in atomic-spin sensing of 1d molecular chains," *ACS Nano*, vol. 16, pp. 16402–16413, 2022.

[14] S. Grimme, J. Antony, S. Ehrlich, and H. Krieg, "A consistent and accurate ab initio parametrization of density functional dispersion correction (DFT-D) for the 94 elements H-Pu," *J. Chem. Phys.*, vol. 132, no. 15, p. 154104, 2010.

[15] S. Grimme, S. Ehrlich, and L. Goerigk, "Effect of the damping function in dispersion corrected density functional theory," *J. Comput. Chem.*, vol. 32, no. 7, pp. 1456–1465, 2011.

[16] J. Cerdá, M. A. Van Hove, P. Sautet, and M. Salmeron, "Efficient method for the simulation of stm images. i. generalized green-function formalism," *Phys. Rev. B*, vol. 56, pp. 15885–15899, Dec 1997.

[17] E. T. R. Rossen, C. F. J. Flipse, and J. I. Cerdá, "Lowest order in inelastic tunneling approximation: Efficient scheme for simulation of inelastic electron tunneling data," *Phys. Rev. B*, vol. 87, p. 235412, Jun 2013.

[18] J. M. Soler, E. Artacho, J. D. Gale, A. García, J. Junquera, P. Ordejón, and D. Sánchez-Portal, "The siesta method for ab initio order- n materials simulation," *Journal of Physics: Condensed Matter*, vol. 14, no. 11, p. 2745, 2002.

[19] J. E. Hasbun and T. Datta, *Introductory Solid State Physics with MATLAB® Applications*. CRC Press, Taylor and Francis Group, 2020.

[20] M. W. Haverkort, M. Zwierzycki, and O. Andersen, "Multiplet ligand-field theory using wannier orbitals," *Physical Review B*, vol. 85, no. 16, p. 165113, 2012.

[21] M. W. Haverkort, G. Sangiovanni, P. Hansmann, A. Toschi, Y. Lu, and S. Macke, "Bands, resonances, edge singularities and excitons in core level spectroscopy investigated within the dynamical mean-field theory," *EPL (Europhysics Letters)*, vol. 108, p. 57004, 12 2014.

[22] M. Retegan, "Crispy: v0.7.3," 2019.

[23] J. C. Slater and J. C. Phillips, "Quantum theory of molecules and solids vol. 4: The self-consistent field for molecules and solids," *Physics Today*, vol. 27, p. 49, 1974.

[24] A. Ferrón, F. Delgado, and J. Fernández-Rossier, "Derivation of the spin Hamiltonians for Fe in MgO," *New J. Phys.*, vol. 17, no. 3, p. 033020, 2015.

[25] R. Rejali, D. Coffey, J. Gobeil, J. W. González, F. Delgado, and A. F. Otte, "Complete reversal of the atomic unquenched orbital moment by a single electron," *npj Quantum Materials*, vol. 5, no. 1, p. 60, 2020.

[26] A. Abragam and B. Bleaney, *Electron Paramagnetic Resonance of Transition Ions*. Oxford University Press, Oxford, 1970.

[27] Z. Zolnierek, "Crystal field parameters in a modified point charge model," *Journal of Physics and Chemistry of Solids*, vol. 45, no. 5, pp. 523–528, 1984.

[28] Z. Dun, X. Bai, M. B. Stone, H. Zhou, and M. Mourigal, "Effective point-charge analysis of crystal fields: Application to rare-earth pyrochlores and tripod kagome magnets $r_3\mathrm{Mg}_2\mathrm{Sb}_3\mathrm{O}_{14}$," *Phys. Rev. Res.*, vol. 3, p. 023012, Apr 2021.

[29] P. Carra, B. T. Thole, M. Altarelli, and X. Wang, "X-ray circular dichroism and local magnetic fields," *Physical Review Letters*, vol. 70, no. 5, pp. 694–697, 1993.

[30] B. T. Thole, P. Carra, F. Sette, and G. van der Laan, "X-ray circular dichroism as a probe of orbital magnetization.," *Physical review letters*, vol. 68, no. 12, pp. 1943–1946, 1992.

[31] D. Weller, J. Stöhr, R. Nakajima, A. Carl, M. Samant, C. Chappert, R. Mégy, P. Beauvillain, P. Veillet, and G. Held, "Microscopic origin of magnetic anisotropy in au/co/au probed with x-ray magnetic circular dichroism," *Physical Review Letters*, vol. 75, no. 20, p. 3752, 1995.

[32] J. Stöhr and H. König, "Determination of spin-and orbital-moment anisotropies in transition metals by angle-dependent x-ray magnetic circular dichroism," *Physical review letters*, vol. 75, no. 20, p. 3748, 1995.

[33] J. Zhang, A. Shchyrba, S. Nowakowska, E. Meyer, T. A. Jung, and M. Muntwiler, "Probing the spatial and momentum distribution of confined surface states in a metal coordination network," *Chem. Commun.*, vol. 50, pp. 12289–12292, 9 2014.

[34] B. Lowe, J. Hellerstedt, A. Matěj, P. Mutombo, D. Kumar, M. Ondráček, P. Jelinek, and A. Schiffrin, "Selective activation of aromatic c–h bonds catalyzed by single gold atoms at room temperature," *Journal of the American Chemical Society*, vol. 144, pp. 21389–21397, 11 2022.

[35] J. Girovsky, J. Nowakowski, M. E. Ali, M. Baljozovic, H. R. Rossmann, T. Nijs, E. A. Aeby, S. Nowakowska, D. Siewert, G. Srivastava, *et al.*, "Long-range ferrimagnetic order in a two-dimensional supramolecular kondo lattice," *Nature communications*, vol. 8, no. 1, p. 15388, 2017.
